# PI3K/mTOR inhibitors promote G6PD autophagic degradation and exacerbate oxidative stress damage to radiosensitize small cell lung cancer

**DOI:** 10.1038/s41419-023-06171-7

**Published:** 2023-10-06

**Authors:** Huan Deng, Yamei Chen, Li Wang, Yibi Zhang, Qingqing Hang, Peijing Li, Peng Zhang, Jing Ji, Hai Song, Ming Chen, Ying Jin

**Affiliations:** 1 Zhejiang Cancer Hospital, Hangzhou Institute of Medicine (HIM), Chinese Academy of Sciences, Hangzhou, Zhejiang 310022 China; 2 State Key Laboratory of Oncology in South China, Guangdong Provincial Clinical Research Center for Cancer, Sun Yat-sen University Cancer Center, Guangzhou, 510060 P. R. China; 3 Institute of Molecular Medicine, Renji Hospital, Shanghai Jiaotong University School of Medicine, Shanghai, 200240 China; 4 Department of Cardiovascular Medicine, The Second Affiliated Hospital of Nanchang University, Nanchang, Jiangxi 331800 China; 5 The MOE Key Laboratory of Biosystems Homeostasis & Protection, Zhejiang Provincial Key Laboratory for Cancer Molecular Cell Biology and Innovation Center for Cell Signaling Network, Life Sciences Institute, Zhejiang University, Hangzhou, Zhejiang 310058 China; 6 United Laboratory of Frontier Radiotherapy Technology of Sun Yat-sen University & Chinese Academy of Sciences Ion Medical Technology Co., Ltd, Guangzhou, China; 7 Zhejiang Key Laboratory of Radiation Oncology, Hangzhou, Zhejiang 310022 China

**Keywords:** Small-cell lung cancer, DNA damage response

## Abstract

Our previous study revealed that PI3K/AKT/mTOR signaling was associated with SCLC radioresistance. SBC2 cells were used as primary radioresistance models, while H446 cells were continuously exposed to ionizing radiation (IR) to develop acquired radioresistance. Cell viability and apoptosis assays were used to investigate synergistic effects of BEZ235/GSK2126458 and IR in vitro, while immunoblotting, metabolite quantitative analysis and bioinformatic analyses were utilized to explore the underlying mechanism. Both genetically engineered mouse models (GEMM) and subcutaneous tumor models were used to confirm the synergistic effect in vivo. Key molecules of PI3K/AKT/mTOR signaling were upregulated after IR, which was correlated with primary radioresistance, and they were more expressed in acquired radioresistant cells. BEZ235/GSK2126458 effectively enhanced the cytotoxic effects of IR. BEZ235/GSK2126458 plus IR elevated γ-H2AX and p-Nrf2 expression, suggesting DNA and oxidative stress damage were intensified. Mechanistically, BEZ235/GSK2126458 plus IR significantly reduced the expression of G6PD protein, the rate-limiting enzyme of the pentose phosphate pathway (PPP). In detail, PI3K/mTOR inhibitors reinforced interaction between G6PD and HSPA8/HSC70, and G6PD was degraded by chaperone-mediated autophagy processes. Their metabolites (NADPH and R-5P) were decreased, and ROS levels were indirectly elevated, both of which exacerbated cell death. PI3K/AKT/mTOR signaling activator, insulin, enhanced SCLC radioresistance, while the synergistic effect of BEZ235/GSK2126458 and IR can be attenuated by N-acetylcysteine, and enhanced by 6-amino niacinamide. GEMM and allograft transplantation assays further confirmed their synergistic effect in vivo. This study provided insights into the connection between PI3K/AKT/mTOR signaling and the PPP underlying radioresistance and provided evidence of mechanisms supporting PI3K/mTOR inhibitors as possible therapeutic strategies to abrogate SCLC radioresistance.

## Background

Small cell lung cancer (SCLC) accounts for ~15% of patients with lung cancer, and is the most recalcitrant lung cancer subtype [1]. SCLC is divided into limited-stage (LS-SCLC) and extensive-stage (ES-SCLC). The standard treatment for LS-SCLC remains concurrent chemoradiotherapy (CCRT) [2, 3]. Although CCRT is effective in early treatment, resistance to chemoradiotherapy always develops in most SCLC patients, followed by progression and relapse [4, 5]. Concerning the low 5-year survival rate in SCLC patients, radioresistance remains a significant clinical challenge. Therefore, it is imperative to find effective strategies to overcome its radioresistance.

Phosphoinositide 3-kinase (PI3K)/protein kinase B (AKT)/mammalian target of rapamycin (mTOR) signaling was upregulated after ionizing radiation (IR) exposure, which promotes tumor cell survival [6]. Recent work from our group showed that PI3K/AKT pathway was enriched according to the whole-exome sequencing (WES) results from eleven treatment-naïve and paired recurrent SCLC patients treated with CCRT [7]. Moreover, PI3K/AKT/mTOR pathway plays pivotal roles in promoting cell proliferation and survival mainly by phosphorylating two downstream effectors, namely ribosomal protein S6 kinase (p70S6K) and eukaryotic translation initiation factor 4E (eIF-4E)-binding protein 1 (4E-BP1) [8].

Glycolysis, oxidative phosphorylation, and the pentose phosphate pathway (PPP) are three primary branches of glucose metabolism, all of which are closely involved in radioresistance [9–11]. High glycolytic levels in tumor cells were found to be significantly correlated with resistance to chemoradiotherapy and aggressive biological behaviors [12–14]. Furthermore, nicotinamide adenine dinucleotide phosphate (NADPH) in the cytoplasm is primarily sourced from the PPP, which helps cells scavenge cellular ROS, maintain the redox balance, and finally elevate the antioxidant capacity of cancer cells [15]. The PPP also provides ribonucleotides to synthesize nucleotides, which helps tumor cells repair DNA damage. This pathway is usually upregulated in rapidly dividing tumor cells in response to DNA damage and oxidative stress triggered by IR, and its dysregulation is closely associated with patient’s prognosis [16–18]. Glucose-6-phosphate dehydrogenase (G6PD) acts as the rate-limiting enzyme of the PPP, and its substrate, glucose-6-phosphate, links glycolysis with the PPP [19, 20]. One recent study showed that PI3K/AKT activation inhibited the degradation of G6PD protein and promoted the PPP through the suppression of tripartite motif-containing 21 (TRIM21), but the crosstalk between PI3K/AKT/mTOR signaling and G6PD requires further elaboration [21].

In this study, we aimed to explore the metabolic rewiring behind radioresistance and to find therapeutic approaches to overcome radioresistance. Pharmacological inhibition of PI3K/AKT/mTOR signaling improved the effectiveness of radiotherapy, including inhibiting cell proliferation, increasing cell apoptosis, and suppressing cell migration. These increased anti-tumor activities were caused by more severe DNA damage triggered by increased ROS levels and decreased NADPH levels. Compared with their parental cells, acquired radioresistant SCLC cells had higher protein levels but lower mRNA expression of G6PD. Inhibition of PI3K/AKT/mTOR pathway reduced the G6PD protein level but elevated its mRNA level. Analysis of the public SCLC dataset showed that patients with lower mRNA expression of G6PD had unfavorable survival. These interesting findings were due to accelerating G6PD degradation through chaperone-mediated autophagy triggered by PI3K/mTOR inhibitors, demonstrating that blocking PI3K/AKT/mTOR pathway was effective in abrogating SCLC radioresistance.

## Materials and methods

### Cell culture and reagents

Three human SCLC cell lines (SBC2, H446 and DMS53) were obtained from Cobioer Corporation and authenticated through short-tandem repeat profiling. Two murine SCLC cell lines derived from the mouse SCLC model (*Cgrp*^*CreER*^*;Trp53*^*f/f*^*;Rb1*^*f/f*^*;Pten*^*f/f*^ (referred to as Cgrp^CreER^;TKO (triple knockout))) were kindly provided by Professor Song Hai (Life Sciences Institute, Zhejiang University). These cell lines were maintained in RPMI-1640 medium (Gibco) supplemented with 10% FBS (Sigma) and 1% penicillin/streptomycin (Sigma).

BEZ235, GSK2126458 and 6-aminonicotinamide (6-AN) were purchased from Selleck Company (Shanghai, China). N-acetylcysteine, MG-132 and insulin were acquired from Beyotime Biotechnology (Shanghai, China). Chloroquine (CQ) was obtained from Sigma Corporation (Shanghai, China), and cycloheximide (CHX) was obtained from MedChemExpress Corporation (Shanghai, China). Anti-phospho-Akt (Ser473), Anti-phospho-Akt (Thr308), anti- Akt (pan), anti–phospho-p70 S6 kinase (Thr389), anti-p70 S6 Kinase (49D7), anti–phospho-4E-BP1 (Ser65), anti-4E-BP1 (53H11), anti-phospho-mTOR (Ser2448), anti-mTOR (7C10), anti-PARP (46D11) and cleaved-PARP (Asp214) antibodies were obtained from Cell Signaling Technology (TX, USA). Anti-G6PD, anti-γ-H2AX, anti-phospho-ATM (Ser1981), anti-phospho-Nrf2(S40), anti-Glut1, anti-SQSTM1/p62 and anti-LC3B antibodies were obtained from Abcam (MA, USA), while anti-phospho-CHK2 (Thr68), anti-beta actin and anti-GAPDH antibodies were purchased from Proteintech (Shanghai, China). Detailed information of antibodies was revealed in Table S1.

### IR

Cancer cells were seeded and allowed to attach overnight. After incubation with BEZ235 and GSK2126458 for 12 h, SCLC cells were treated with a single fraction (2 to 6 Gy) of radiotherapy delivered by a small animal radiation research platform (SARRP, Xstrahl Inc., Suwanee, GA, USA). In addition, Cgrp^CreER^;TKO mice were anesthetized using 1.25% tribromoethanol and immobilized on the mouse bed of the Xstrahl SARRP, where the source-to-surface distance was set at 333 mm and the irradiation field was 10 mm × 10 mm. Four IR fractions (6 Gy per treatment) were delivered to the mouse thorax over 2 weeks with a dose rate of 6 Gy/min.

### Establishment of acquired radioresistant SCLC cells

SCLC cells were seeded in a 75cm^2^ culture flask. After SCLC cells adhered to the wall, a single dose of 2 Gy was irradiated to the cells. The irradiated cells were put in the incubator, and the growth status of the cells was closely observed. After the cell state gradually recovered, cells were then irradiated with the next single dose of 2 Gy. Repeatedly, SCLC cells were irradiated with a 60 Gy dose of radiation in total. The surviving cells were believed to be cells with acquired radioresistance.

### Cell viability assay

Cells were plated into 96-well plates and allowed to attach overnight before exposure to BEZ235/GSK2126458 or IR. After 72 h of treatment, the medium was removed, and a new medium containing 10% cell counting kit 8 (CCK8, APExBIO) was added. After culture for one to 2 h at 37 °C, the absorbance was measured at 450 nm as previously described [7]. Normalized transformed dose-response curves were obtained and analyzed by GraphPad Prism (GraphPad Software Inc.).

### Colony formation assay

A colony formation assay was utilized to evaluate the radiosensitivity of SCLC cells. Briefly, cells were plated into 6-well plates and treated with different treatments. Chemical reagents were removed at 72 h after IR, and the cells were cultured for 8–12 days to develop colonies and stained using Giemsa stain (Solarbio). Colonies with >50 cells were used to calculate the survival fraction.

### Immunoblotting

Protein samples were mixed with 5x SDS‐PAGE loading buffer and boiled at 100 °C for 10 min. After electrophoresis, separated proteins were transferred to PVDF blotting membranes, and membranes were blocked with 5% BSA at room temperature for 90 min. Next, membranes were incubated with primary antibodies (Table S1) at 4 °C for 16 h, washed using TBS containing 0.1% Tween 80 (TBST) for 8 min each time at room temperature and incubated with secondary antibodies at room temperature for 75 min. After three washes using TBST, immunoreactive bands were visualized using enhanced chemiluminescent reagent (ECL, Millipore Biotechnology). Relative protein levels were quantified by ImageJ software.

### Quantitative real-time PCR

Total RNA was extracted using TRIzol reagent (Accurate Biology). Reverse transcription of total RNA was performed using *EVO M-MLV* Reverse Transcriptase (Accurate Biology). Quantitative real-time PCR was carried out using SYBR Master Mixture (APExBIO) and 7500 Fast Real‐time PCR system (Thermo Fisher Scientific). GAPDH gene levels were utilized for relative quantification. The primers are available in Table S2. The results were calculated using the 2^-ΔΔCt^ method to calculate the relative expression level of the target gene at the mRNA level.

### Immunofluorescence (IF) assay

After three washes using phosphate-buffered saline (PBS, Beyotime Biotechnology), cells plated on 12-well plates were fixed using 4% paraformaldehyde for 30 min. Subsequently, cells were penetrated using Triton X-100 (Beyotime Biotechnology) for 30 min and blocked using 3% BSA at room temperature for 45 min. Next, the primary antibodies were applied at 4 °C for 16 h and washed using PBS three times. Afterward, cells were incubated with Alexa Fluor 488/647-labeled secondary antibodies (Abcam) in dark for 60 min. Finally, the cells were stained with DAPI dihydrochloride (DAPI) to visualize the nucleus, and images were obtained using a fluorescence microscope (Olympus, Tokyo, Japan). Three independent fields were randomly chosen under the microscope, and the mean fluorescence intensity (MFI) was calculated by ImageJ software.

### Hoechst staining

Cells treated with PI3K/mTOR inhibitors combined with or without IR were harvested 48 h after radiotherapy, and stained using Hoechst 33342 staining solution (Beyotime Biotechnology) for 10 min. Densely stained cells were regarded as apoptotic cells according to the manufacturer’s instructions, and images were obtained using a fluorescence microscope (Olympus, Tokyo, Japan).

### Annexin V/propidium iodide staining

Cells treated with PI3K/mTOR inhibitors combined with or without IR were digested using trypsin solution without EDTA 48 h after radiotherapy, and cell pellet were harvested by centrifugation. Afterwards, cells were stained with Annexin V-FITC and propidium iodide (PI) for 5 min using the Annexin-V-FITC/PI apoptosis kit (MULTI SCIENCES) according to the manufacturer’s instructions. Stained cells were analyzed with flow cytometry using fluorescence emission at 488 nm and 617 nm. Cells with only green fluorescence were regarded as early apoptotic cells, while cells with both green and red fluorescence were regarded as late apoptotic cells.

### Measurement of ROS and NADPH production

Cells were stained with MitoSOX Red (Thermo Fisher Scientific) for 10 min and analyzed with flow cytometry using fluorescence emission at 488 nm. To evaluate the production of ROS MFI values in the live-cell gate were calculated using SP6800 Spectral Analyzer (Sony Biotechnology). When double peaks appeared, the right peak was believed to be the positive peak of ROS. The ratio of positive cells was calculated to appraise the level of ROS.

NADPH levels were measured using the NADP^+^/NADPH assay (Beyotime Biotechnology) following the manufacturer’s recommendations. In brief, the NADP^+^/NADPH extraction solution was added to 2 * 10^6^ cells to obtain NADP^+^/NADPH. The samples were heated at 60 °C for 30 min to remove NADP^+^. Subsequently, the chromogenic agent was added to the samples for 30 to 45 min, and the absorbance was measured at 450 nm.

### Metabolite quantitative analysis

After different treatments, cells were digested using 0.25% trypsin and centrifuged to acquire cell precipitation. The quantitative metabolomics of energy metabolism was assessed by Metabo-Prolife Company (Shanghai, China) to obtain the levels of kinds of metabolites in cells treated with different treatments. The final level of all metabolites was adjusted to the protein concentration.

### RNA-seq analysis

After extraction from SCLC cells, total RNA was processed at Beijing Genomics Institution (BGI) for RNA-seq with the DNBSEQ platform. Approximately 40 million reads per sample were acquired.

The edgeR package of R (4.2.1) was utilized to perform differential gene expression analysis of RNA-Seq datasets to acquire differentially expressed genes (DEGs) between the acquired radioresistant cells and their parental cells. To reduce the false discovery rate, the *P*-value was adjusted with the Benjamini–Hochberg correction. |logFC > 1 and adj. *P* < 0.05 were used to select DEGs. Gene Ontology (GO) and Kyoto Encyclopedia of Genes and Genomes (KEGG) pathway analyses of DEGs were carried out using the clusterProfiler package [22, 23], and *P* < 0.05 was considered a significant difference.

### Bioinformatic analysis

GSE211118 was used to explore the biological effect in lung cancer cells treated with or without IR [24]. To identify the role of G6PD in radioresistance, relevant datasets from public platforms were downloaded. GSE207002 and GSE210411 were used to explore the underlying molecular mechanisms behind the acquired radioresistance. RNA-seq data and clinical information of 77 SCLC patients from the cBioPortal platform were acquired and analyzed to determine the prognostic roles of oxidative stress-related genes [25]. In addition, GSE31210 was utilized to investigate the difference in G6PD between NSCLC and SCLC. To analyze the roles of oxidative stress-related genes in primary radioresistance, a dataset of SCLC cell lines from the Cancer Cell Line Encyclopedia (CCLE) platform was obtained for further analysis [26].

### Measurement of the half-life of G6PD protein

SBC2 cells were treated with either CHX alone, CHX + PI3K/mTOR inhibitors, or CHX + PI3K/mTOR inhibitors + CQ. Protein samples were obtained at 0, 4, 8, 12 and 16 h after the indicated treatments. G6PD and GAPDH protein levels were quantified by ImageJ software, and GAPDH levels were utilized to measure the relative level of G6PD. The G6PD level at the four timepoints was relatively quantified using the value at 0 h. These outcomes represented the fraction of remaining G6PD protein, and the time corresponding to 50% was the half-life of G6PD protein.

### Co-immunoprecipitation (Co-IP) assay

According to instructions provided by the manufacturer, the whole cell lysates of SBC2 cells with G6PD overexpression were obtained and centrifuged at 10,000 xg for 15 min. Supernatant was incubated anti-Flag antibody (mouse) or anti-IgG antibody (mouse) for 12 h at 4 °C. Subsequently, samples were washed three times with TBS. To identify proteins interacted with G6PD, mass spectrometric analysis was conducted by PTM BIOLAB (Hangzhou, China) to identify proteins interacted with G6PD. Afterwards, samples were mixed with SDS‐PAGE loading buffer and boiled at 100 °C for 10 min, and immunoblotting assay were performed as previously described. Relevant antibodies were shown in Table S1.

### Animal experiments

The genetically engineered mouse model (GEMM), Cgrp^CreER^;TKO, was kindly provided by Professor Song Hai (Life Sciences Institute, Zhejiang University) [27]. Corn oil was used to prepare a tamoxifen (Aladdin) solution at 20 mg/ml. To induce the formation of SCLC, intraperitoneal injections with tamoxifen (2 mg/25 g (body weight)) were performed three times. Mice were randomized into four groups 30 days after tamoxifen injection: negative control; BEZ235 (40 mg/kg/day); IR (6 Gy * 4) and IR + BEZ235. BEZ235 was administered daily by oral gavage, and four IR fractions (6 Gy per treatment) were performed in 2 weeks. In the combination therapy group, mice were administered BEZ235 at 2 h before IR exposure.

Four-week-old female BALB/c athymic mice were provided by the SLAC laboratory (Shanghai, China). Mouse SCLC (1 * 10^7^) cells were injected subcutaneously into the right flank to form allograft tumors. Mice were randomized into four groups when tumor volume reached about 300 mm^3^: control (Vehicle); BEZ235 (40 mg/kg/day); IR (6 Gy * 4) and IR + BEZ235. Tumor volume (V) was calculated every three days using the equation: *V* = *L* x *W*^2^/2 (*L* = length, *W* = width). When tumor volume reached 1500 mm^3^, the mice were euthanized, and the allograft tumors were isolated and photographed. Afterward, the tumors were divided into two parts, one part was fixed and embedded in 10% paraformaldehyde for IHC, while the other one was reserved in liquid nitrogen. All animal care and experimental procedures used in the study received approval from the Animal Ethics Committee of Zhejiang Cancer Hospital (2022–09–001).

### Hematoxylin-eosin (HE) staining and immunohistochemistry (IHC) assay

Mouse lungs were perfused using PBS and fixed overnight in 10% paraformaldehyde. Lungs were dehydrated, embedded, cut into slices, and stained with hematoxylin solution for 5–8 min. Subsequently, these slices were placed into 1% alcohol hydrochloric acid for differentiation for 3–5 s and changed back to blue. Neutral gum was used for sealing, and the slices were observed under the microscope.

Allograft tumors were stained with anti-Ki67 antibodies (1:200) and counterstained with hematoxylin. IHC images were captured under a microscope. Three independent, random fields were selected for semiquantitative analysis using Image J software. The average optical density (AOD) was measured using the formula AOD = Integrated optical density (IOD)/Area.

### Statistical analysis

Cellular experiments were performed in triplicate, and the data obtained in this study were processed and analyzed by GraphPad Prism (GraphPad Software Inc.). Relevant data were expressed as mean+SD. Student’s two-sided *t*-test was used to compare the difference between the two groups, while the one-way analysis of variance (ANOVA) was used to compare the difference between more than two groups. *P* < 0.05 was regarded as statistical significance.

## Results

### The impact of IR on PI3K/AKT pathway, and the influence of blocking this pathway on glucose metabolism in SCLC cells

To explore the biological effect of IR, RNA-seq analysis was performed by comparing the expression profile of SBC2 cells treated with or without IR. GO analysis revealed that regulation of transcription from RNA polymerase II promoter, apoptotic process and cellular response to DNA damage stimulus were significantly enriched, while KEGG pathway analysis showed that pathways in cancer, transcriptional misregulation in cancer, and cellular senescence (Fig. S1A, B). The GSE211118 dataset also compared the expression profiles in A549 and H446 cells treated with or without X-ray radiation. A total of 5398 DEGs were obtained in A549 cells, and GO analysis revealed that DNA repair, apoptotic process and cellular response to DNA damage stimulus and cellular response to oxidative stress were significantly enriched, most of which were accord with the effect of IR on killing tumor cells (Fig. S1C). KEGG pathway analysis showed that metabolic pathways, pathways in cancer and PI3K/AKT signaling pathway were significantly enriched, suggesting that these pathways possibly were involved in cell survival and proliferation after exposure to IR (Fig. S1D). Similar results were found in H446 cells (Fig. S1E, F).

Previously, we showed that PI3K/AKT signaling was significantly enriched in recurrent SCLC samples, and this signaling was associated with therapeutic resistance [7]. To further investigate the role of PI3K/AKT pathway in SCLC radioresistance, we compared the PI3K/AKT pathway-related proteins in cells treated with IR and those without IR exposure. According to the outcomes of immunoblotting, these proteins related to PI3K/AKT signaling were significantly upregulated 24 h after IR exposure in the three SCLC cell lines (Fig. S2A-C). Quantitative analysis revealed that the upregulation of these proteins in SBC2 cells was more significant than that in H446 and DMS53 cells (Fig. S2D). Because multiple SCLC cell lines did not form colonies, CCK8 assay was used to detect the radiosensitivity of SBC2, H446 and DMS53 cells as shown in the study [28]. SBC2 cells had the lowest sensitivity to IR compared with H446 and DMS53 cells (Fig. S2E), suggesting that the higher activation of PI3K/AKT signaling after IR exposure probably promoted cell survival and contributed to primary radioresistance. Therefore, SBC2 was selected as the primary radioresistant cell model, whereas H446 was chosen to develop acquired radioresistance by irradiating cells with multiple fractionated doses of X-rays.

BEZ235 and GSK2126458, dual PI3K/mTOR inhibitors, were selected in our experiments to completely block the PI3K/AKT/mTOR pathway. The inhibition of PI3K/AKT/mTOR signaling caused by the two inhibitors had a significant impact on glucose metabolism. Glucose intake was significantly reduced at 24 h after exposure to BEZ235/GSK2126458, and the production of lactic acid was also decreased (Fig. S2F, G and Fig. S3A, B).

### The influence of the combination of IR and dual PI3K/mTOR inhibitors on the proliferation and apoptosis of SCLC cells

**Fig. 1:**
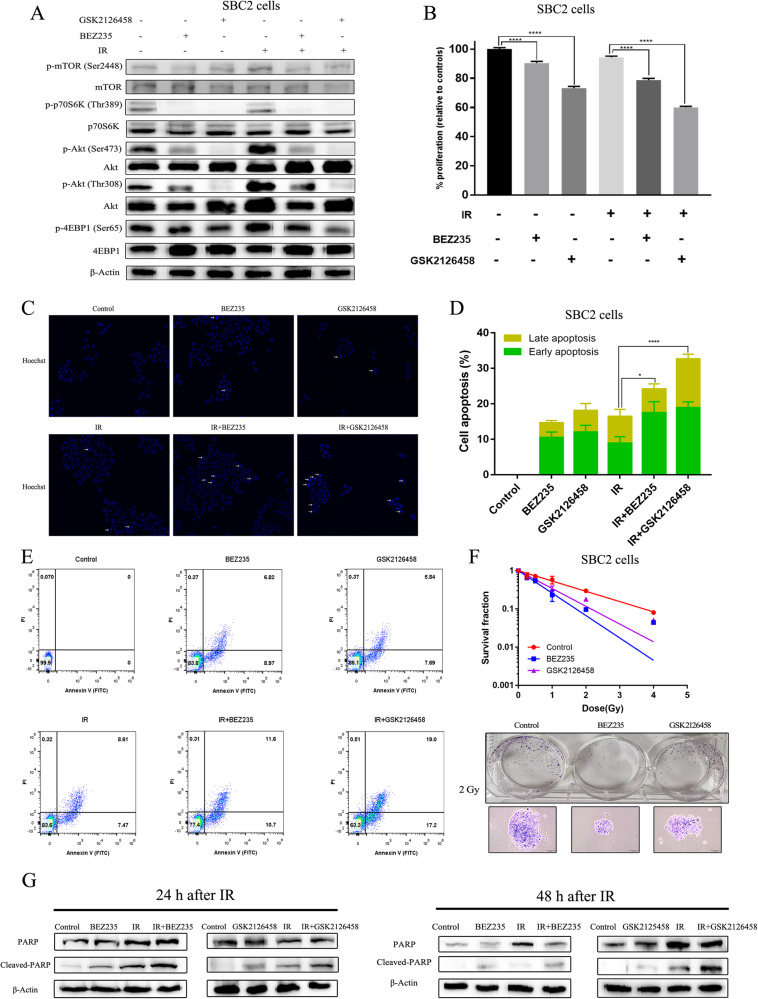
PI3K/mTOR inhibitors overcome the primary radioresistance of SBC2 cells. **A** The time (12 h before IR) and concentration (50 nM) of PI3K/mTOR inhibitors could effectively inhibit PI3K/AKT/mTOR signaling. **B** The cell proliferation of cells treated with BEZ235 and GSK2126458 combined with or without IR were determined by CCK8 assay at 72 h after 6 Gy of X-ray irradiation. **C** Apoptotic cells were revealed by Hoechst staining. **D**, **E** The cell apoptosis of cells treated with PI3K/mTOR inhibitors combined with or without IR were detected by Annexin V/PI staining. **F** The survival fraction of SBC2 cells treated with IR combined with or without PI3K/mTOR inhibitors were determined by colony formation assay, and the number and size of SBC2 cell clones were detected under 2 Gy radiation exposure. **G** The expression of PARP and Cleaved-PARP across various groups were detected 24 h and 48 h after radiotherapy by immunoblotting.

BEZ235 and GSK2126458 were added 12 h before IR exposure. Their inhibitory effects were investigated in three SCLC cell lines (SBC2, DMS53, and H446) at 24 h, 48 h, and 72 h after the addition of PI3K/mTOR inhibitors. Both BEZ235 and GSK2126458 showed strong antitumor activity, with a low 50% inhibitory concentration (IC50) (nM class) in three human SCLC cell lines (Fig. S2H, I and Fig. S3C, D). Furthermore, the dose of PI3K/mTOR inhibitors (50 nM) effectively blocked PI3K/Akt/mTOR pathway in three human SCLC cell lines, with the significantly decreased expression of phospho-Akt (Ser473 and Thr308), phospho-mTOR (Ser2448), phospho-4EBP1 (Ser65), and phospho-p70S6K (Thr389) (Fig. 1A and Fig. S3E, F).

Compared to the utilization of either modality alone, BEZ235/GSK2126458 plus IR had apparently stronger cytotoxic effects in three human SCLC lines, with enhanced inhibition of cell proliferation and increased apoptosis. According to the results of the CCK8 assay, proliferation capacity of SBC2, DMS53, and H446 cells were significantly inhibited in cells treated with the combination of BEZ235/GSK2126458 with IR compared with the utilization of either modality alone (Fig. 1B and Fig. S3G, H). The Annexin V/PI staining assay revealed that compared with the BEZ235/GSK2126458 and IR groups, there were more apoptotic cells (including early apoptosis and late apoptosis) in the BEZ235/GSK2126458 plus IR group (Fig. 1C–E and Fig. S4A, B). In detail, the mean apoptosis percentage of SBC2 cells treated with BEZ235 was 14.6%, and the percentage of cells treated with IR was 16.4% whereas the percentage of cells treated with BEZ235 plus IR was increased to 24.1%. Similarly, the mean apoptosis percentage of SBC2 cells treated with GSK2126458 was 18.0% while the percentage of cells treated with GSK2126458 plus IR was increased to 32.6%. In addition, colony formation assays showed that both BEZ235 and GSK2126458 could sensitize SBC2 cell to radiation, with the decreased number and size of clones in the combination group (Fig. 1F). Compared to cells treated with BEZ235/GSK2126458 alone and IR alone, cleaved-PARP expression was significantly elevated 24 and 48 h after IR when treated with both BEZ235/GSK2126458 and IR (Fig. 1G and Fig. S4C, D). Although the combination of GSK2126458 and IR demonstrated short-term anti-tumor effectiveness compared to BEZ235 and IR based on CCK8 assay results, BEZ235 plus IR appeared to exhibit superior long-term therapeutic effects, as revealed by clone-forming assay.

**Fig. 2:**
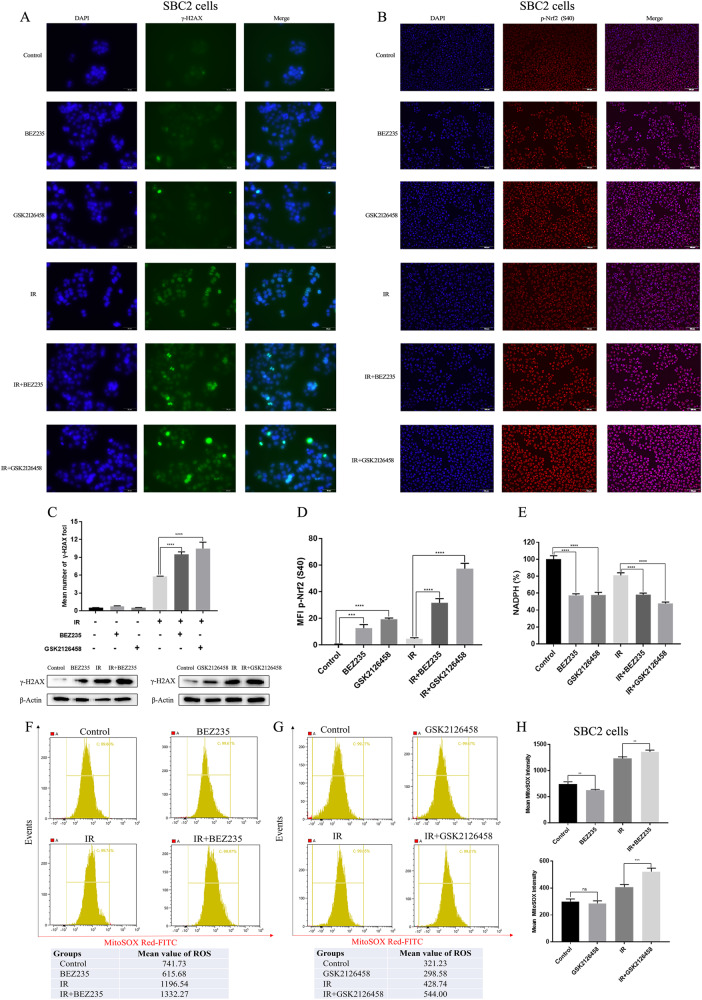
PI3K/mTOR inhibitors combined with IR led to more serious DNA damage and more serious oxidative stress compared with the utilization of either modality alone. **A** γ-H2AX expression in cells treated with PI3K/mTOR inhibitors combined with or without IR were detected by immunofluorescence. **B** The expression of p-Nfr2 (S40) in cells treated with PI3K/mTOR inhibitors combined with or without IR were detected by immunofluorescence. **C** γ-H2AX expression in cells treated with PI3K/mTOR inhibitors combined with or without IR were quantitatively analyzed. **D** The level of p-Nfr2 (S40) in cells treated with PI3K/mTOR inhibitors combined with or without IR were quantitatively analyzed. **E** NADPH level in cells treated with PI3K/mTOR inhibitors combined with or without IR was detected. **F**–**H** ROS level of SBC2 cells across different groups was detected using MitoSOX Red, and the mean level of ROS were calculated.

To further investigate the severity of DNA damage across various groups, γ-H2AX, a molecular marker of DNA damage, was detected using immunoblotting and IF assays. Compared to the utilization of either modality alone, the level of γ-H2AX was significantly elevated in cells treated with BEZ235/GSK2126458 plus IR (Fig. 2A, C and Fig. S4E-H). Besides, p-CHK2 and p-ATM are the markers of DNA damage, and we detected their expression across various groups 45 min and 90 min after radiotherapy. The combination group had higher expression of p-CHK2 and p-ATM compared with monotherapy groups (Fig. S4I). These findings demonstrated that cell apoptosis and DNA damage were more severe in cells treated with PI3K/mTOR inhibitors and IR.

### IR combined with dual PI3K/mTOR inhibitors alters redox homeostasis

Nuclear factor E2-related factor 2 (Nrf2) is a crucial antioxidant transcription factor that protects cells from oxidative stress, and phosphor-Nrf2 (p-Nrf2) quickly accumulates in the cell nucleus to enhance the expression of antioxidant response elements (AREs) under oxidative stress damage. Cells treated with BEZ235/GSK2126458 plus IR had higher expression of p-Nrf2 than the utilization of either modality alone (Fig. 2B, D). Reactive oxygen species (ROS) usually caused DNA damage and oxidative stress damage, so the cellular levels of ROS and NADPH were detected after IR exposure. We found a remarkable decrease in cellular NADPH in cells treated with BEZ235/GSK2126458 regardless of IR exposure, suggesting that the antioxidant capacity of SCLC cells was notably impaired by dual PI3K/mTOR inhibitors (Fig. 2E). Afterward, cells treated with BEZ235/GSK2126458 plus IR had a higher level of ROS than cells treated with BEZ235/GSK2126458 alone or IR alone (Fig. 2F-H). These results indicated that oxidative stress damage was significantly exacerbated in cells treated with PI3K/mTOR inhibitors combined with IR.

### Comparison between acquired radioresistant SCLC cells and their parental cells

**Fig. 3:**
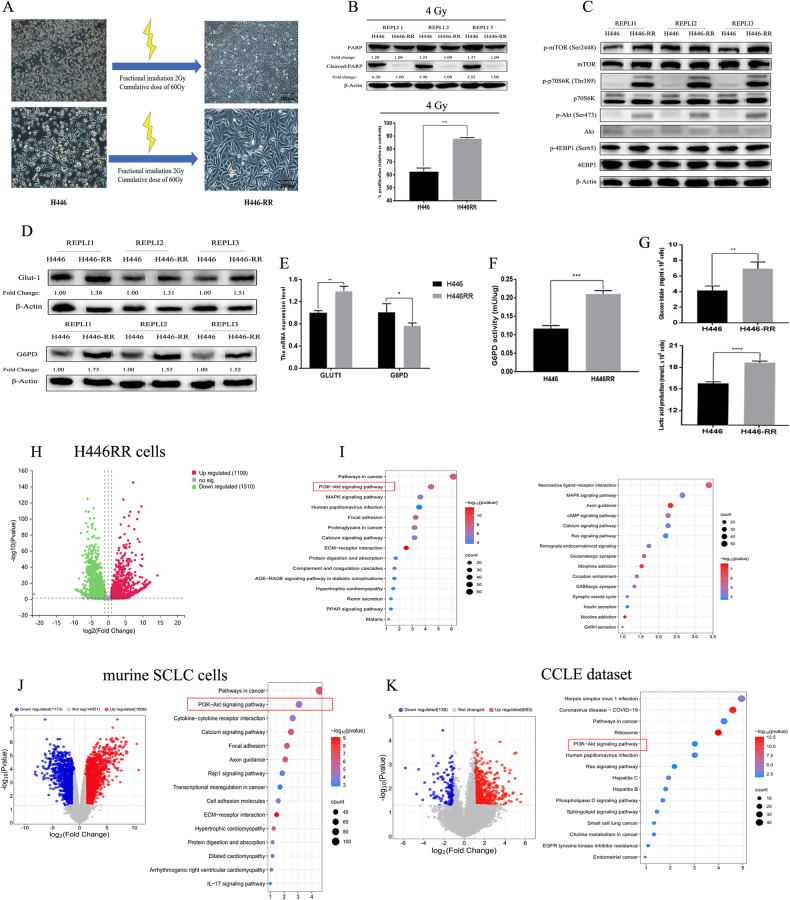
The difference between acquired radioresistant SCLC cells and its parental cell line H446. **A** The process of establishing SCLC cells with the acquired radioresistance was clearly shown. **B** The successful establishment of SCLC cells with the acquired radioresistance was validated. Under the exposure of 4 Gy, the expression of Cleaved-PARP and PARP were determined by immunoblotting and cell proliferation were determined by CCK8 assay. **C** The expression of key proteins related to PI3K/AKT/mTOR pathway between H446RR and H446 were determined by immunoblotting. **D**, **E** The protein and mRNA level of G6PD and GLUT1 between H446RR and H446 cells were determined by immunoblotting and qPCR. **F** G6PD activity between H446RR and H446 were determined. **G** Glucose intake and lactic acid production between H446RR and H446 were determined. **H**, **I** Differential expression analysis was carried out in the RNA-seq dataset between H446RR and H446 cells, and KEGG pathway analysis of these DEGs was performed. **J** Differential expression analysis was carried out in the RNA-seq dataset between acquired radioresistant murine SCLC cells with tdTomato and their parental cells, and KEGG pathway analysis of these DEGs was performed. **K** Differential expression analysis was carried out in CCLE datasets between primary radioresistant and radiosensitive SCLC cells, and KEGG pathway analysis of these DEGs was performed.

To investigate the potential mechanisms behind the acquired radioresistance of SCLC cells, we first established acquired radioresistant H446RR cells by irradiating H446 cells with fractionated doses of X-rays (2 Gy/time) and total doses (60 Gy). The cellular morphology of H446RR cells was completely different from that of their parental H446 cells, with increased cell size and grains (Fig. 3A). When exposed to IR (4 Gy), H446RR cells had less proliferation suppression and decreased cleaved-PARP levels than its parental cell line, suggesting the successful establishment of acquired radioresistance (Fig. 3B). Compared with parental H446 cells, H446RR cells had higher activation of PI3K/AKT/mTOR signaling pathway (Fig. 3C), which further validated our previous WES findings. In addition, the mRNA and protein levels of GLUT1 and G6PD were compared between H446 and H446RR cells, and the mRNA and protein levels of GLUT1 were increased in H446RR (Fig. 3D, E). Interestingly, G6PD protein was upregulated in H446RR cells compared with their parental H446 cells, whereas G6PD mRNA level was decreased in H446RR cells. One recent study using quantitative proteomic analysis demonstrated that G6PD protein was significantly upregulated in radioresistant CNE1 cells [29]. Furthermore, G6PD activity was notably elevated in H446RR cells compared with H446 cells, revealing the upregulation of PPP in H446RR cells (Fig. 3F). Glucose uptake and lactic acid production were also enhanced in H446RR cells (Fig. 3G).

To further explore the potential mechanism behind acquired radioresistance, RNA-seq analysis was performed by comparing the expression profiles of H446RR and H446. The differential expression analysis demonstrated that 1109 upregulated DEGs and 1510 downregulated DEGs were acquired (Fig. 3H). KEGG pathway analysis of these upregulated DEGs showed that PI3K/AKT pathway was significantly enriched (Fig. 3I). Besides, murine SCLC cells with acquired radioresistance was also established using fractionated doses of X-rays, and RNA-seq analysis was carried out by comparing the expression profiles of acquired radioresistant murine SCLC cells and their parental cells. 1606 upregulated DEGs and 1173 downregulated DEGs were obtained, and KEGG pathway analysis of these DEGs also revealed that PI3K/AKT pathway was significantly enriched (Fig. 3J). Furthermore, one study divided SCLC cell lines into primary radioresistant and radiosensitive cell lines according to their radiosensitivity [28]. Differential expression analysis of these cell lines was carried out [30], and 817 DEGs were obtained. The KEGG pathway analysis of these DEGs showed that PI3K/AKT pathway was also significantly enriched (Fig. 3K). In addition, we found two similar datasets in GEO, both of which compared the differences between acquired radioresistant cells and parental cells at the transcriptional level. Differential expression analysis of GSE207002 that compared U87MG cells and their radioresistant cell lines was conducted, and KEGG pathway analysis of DESs revealed that PI3K/AKT pathway was also significantly enriched (Fig. S5A). Similar outcomes were found in GSE210411, which compared MCF-7 cells and their acquired radioresistant cells (Fig. S5B). One recent analysis also compared the transcriptomes of radiosensitive and radioresistant patient-derived organoids in colorectal cancer, and identified that PI3K/AKT pathway was significantly enriched in organoid lines [31]. These consistent findings suggested that upregulation of PI3K/AKT/mTOR signaling probably promoted radioresistance in cancers. Furthermore, we investigated the differences in glucose metabolism between H446RR cells and their parental H446 cells using targeted metabolomics. Compared with their parental H446 cells, the three branches of glucose metabolism (glycolysis, the PPP and TCA cycle) were remarkably upregulated in H446RR cells (Fig. S5C-F).

### Dual PI3K/mTOR inhibitors abrogate the acquired radioresistance in SCLC

**Fig. 4:**
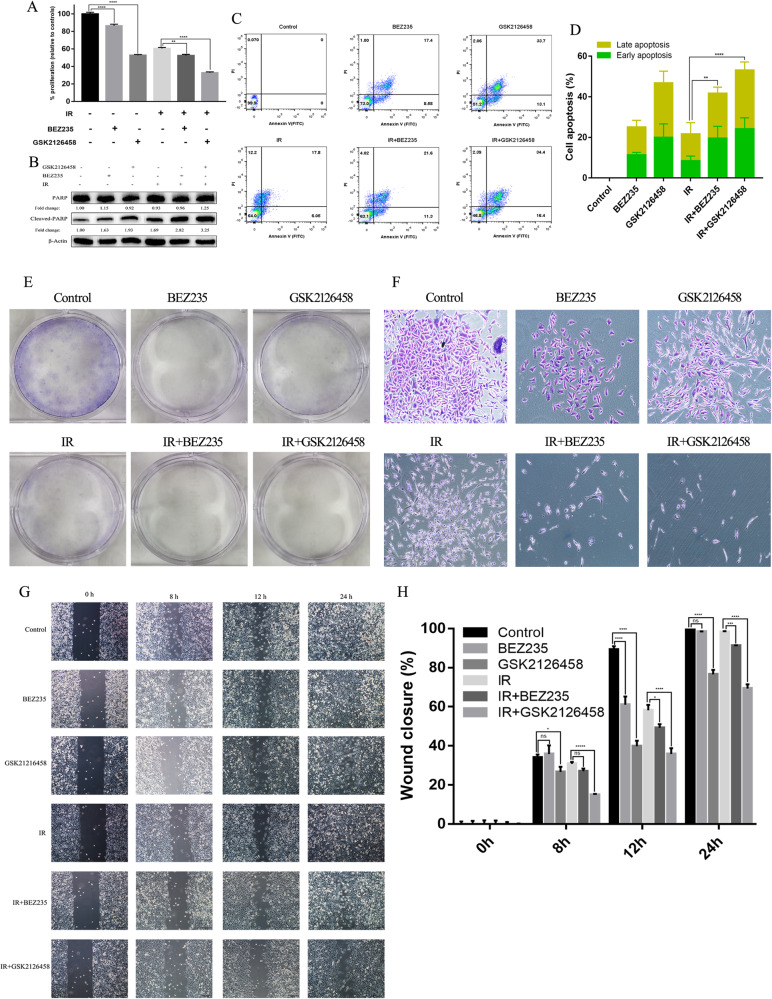
PI3K/mTOR inhibitors increased the anti-tumor effect of IR in H446RR cells. **A** The cell proliferation of cells across various groups were determined by CCK8 assay at 72 h after 6 Gy of X-ray irradiation. **B** The expression of PARP and Cleaved-PARP across various groups were determined by immunoblotting. **C**, **D** The apoptosis of H446RR cells in different groups were detected by Annexin V/PI staining. **E**, **F** The growth of H446RR cells in different groups were detected using Giemsa stain, although H446RR cells typically failed to form colonies when a single cell existed. **G**, **H** The migration of H446RR cells across various treatments were determined at 8 h, 12 h and 24 h by wound healing assay.

BEZ235 and GSK2126458 could effectively improve the anti-cancer activity of IR even in SCLC cells with acquired radioresistance. BEZ235/GSK2126458 increased the inhibiting ability of cell proliferation caused by IR (Fig. 4A). Compared with the utilization of either modality alone, cleaved-PARP expression was significantly increased in cells treated with the combination of BEZ235/GSK2126458 and IR (Fig. 4B). Similarly, the apoptosis rate of H446RR cells (including early apoptosis and late apoptosis) was higher in cells treated with BEZ235/GSK2126458 plus IR. In detail, the mean apoptosis percentage of H446RR cells treated with BEZ235 was 25.1%, and the percentage of cells treated with IR was 21.7% whereas the percentage of cells treated with BEZ235 plus IR was increased to 41.7%. Similarly, the mean apoptosis percentage of H446RR cells treated with GSK2126458 was 46.8% while the percentage of cells treated with GSK2126458 plus IR was increased to 53.1% (Fig. 4C, D). Moreover, although H446RR cells typically did not form colonies when a single cell existed, we also utilized Giemsa stain to reveal the growth and proliferation of cells. The number of H446RR cells were significantly decreased in group treated with PI3K/mTOR inhibitors plus IR, compared to cells treated solely with PI3K/mTOR inhibitors or IR alone (Fig. 4E, F). Based on the wound healing assay results, the combination group demonstrated a slower rate of wound closure than monotherapy groups (Fig. 4G, H). According to the above findings, the anti-tumor effect of IR was improved by BEZ235/GSK2126458 even in acquired radioresistant cells.

**Fig. 5:**
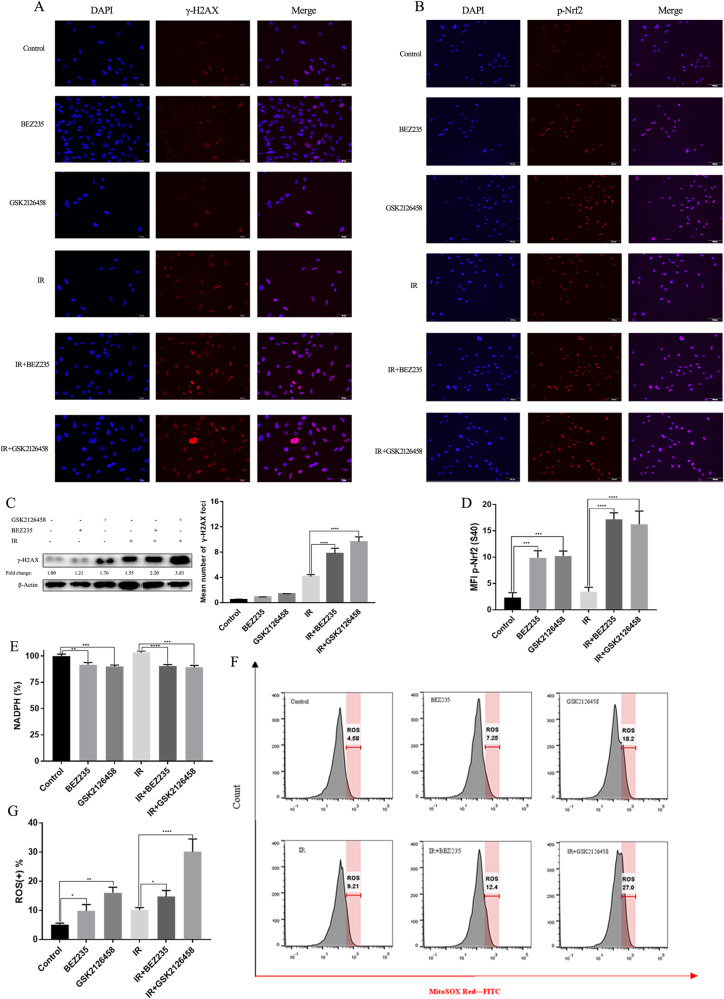
PI3K/mTOR inhibitors intensified DNA damage and oxidative damage caused by IR in H446RR cells. **A** *γ*-H2AX expression across various treatments were determined by immunofluorescence. **B** The expression of p-Nfr2 (S40) across different treatments were determined by immunofluorescence. **C** γ-H2AX level across various treatments was quantitatively analyzed, and γ-H2AX expression were detected by immunoblotting. **D** The p-Nfr2 (S40) expression across different groups was quantitatively analyzed. **E** NADPH level in cells treated with PI3K/mTOR inhibitors combined with or without IR was detected. **F**, **G** ROS level of H446RR cells across different groups was detected using MitoSOX Red, and the mean level of ROS were obtained.

The level of DNA damage in cells was also assessed after IR exposure, and immunoblotting and IF assays were used to determine the expression of γ-H2AX in cells treated with different modalities. γ-H2AX was significantly upregulated in cells treated with PI3K/mTOR inhibitors and IR, revealing that a higher level of DNA damage existed in the combination group (Fig. 5A, C). We also observed p-Nrf2 expression in cells treated with different modalities using IF assay, and p-Nrf2 was significantly increased in cells exposed to BEZ235/GSK2126458 and IR compared with cells exposed to BEZ235/GSK2126458 alone or IR alone, suggesting that oxidative stress in the combination group was obviously intensified (Fig. 5B, D). Furthermore, the cellular levels of ROS and NADPH after IR were detected in H446RR cells treated with different modalities. BEZ235/GSK2126458 reduced cellular NADPH levels in H446RR cells with or without IR exposure (Fig. 5E). Compared with cells exposed to BEZ235/GSK2126458 alone or IR alone, there was a significant increase in ROS levels in H446RR cells treated with BEZ235/GSK2126458 and IR (Fig. 5F). The above results suggested that intensified oxidative stress in the combination group was obviously intensified.

### Bioinformatic analysis of the G6PD radioresistance effect

**Fig. 6:**
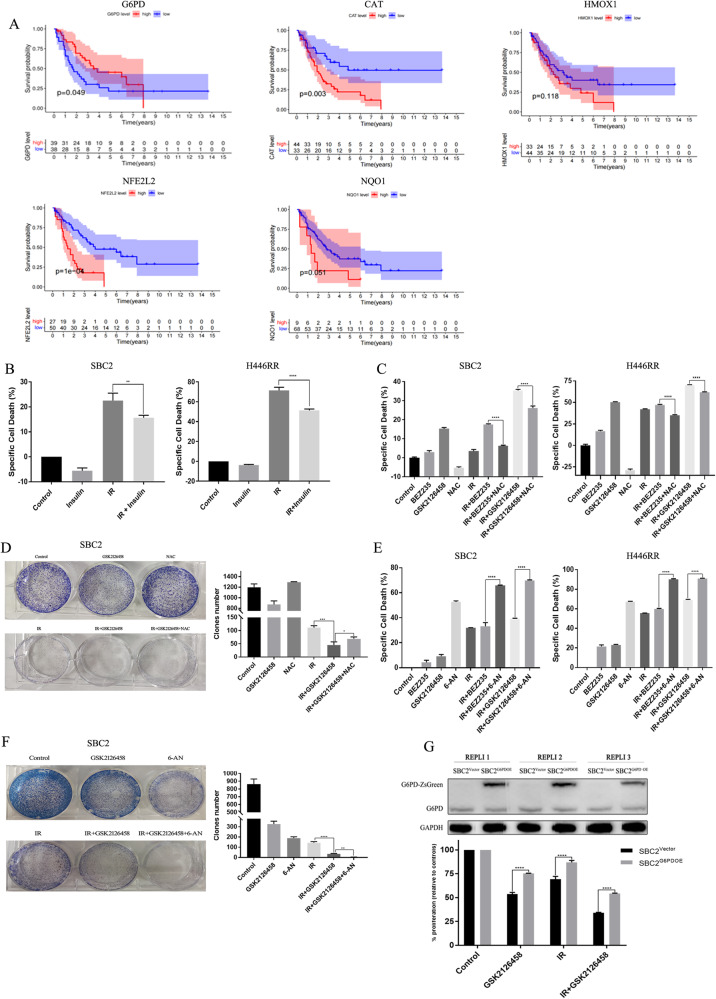
Whether PI3K/AKT/mTOR signaling, ROS and G6PD contributed to SCLC radioresistance were validated. **A** The correlation between oxidative stress-related genes, including G6PD, CAT, HMOX1, NFE2L2 and NQO1, and overall survival (OS) among SCLC patients. **B** The specific cell death of SBC2 and H446RR cells treated with IR plus insulin were determined by CCK8 assay. **C** The specific cell death of SBC2 and H446RR cells treated with PI3K/mTOR inhibitors, IR and NAC were determined by CCK8 assay. **D** The cell growth of SBC2 cells treated with PI3K/mTOR inhibitors, IR and NAC were determined by clone formation assay. **E** The specific cell death of SBC2 and H446RR cells treated with PI3K/mTOR inhibitors, IR and 6-AN were determined by CCK8 assay. **F** The cell growth of SBC2 cells treated with PI3K/mTOR inhibitors, IR and NAC were determined by clone formation assay. **G** The cell proliferation ability cells in mock-vehicle groups or cells transfected with G6PD overexpression vector were detected by CCK8 assay.

To further explore the inconsistency in G6PD between mRNA and protein levels, we analyzed several datasets from the public platform. First, patients with SCLC from the cBioPortal platform were divided into G6PD^high^ and G6PD^low^ groups according to the mRNA level of G6PD, and an obvious difference in overall survival was detected between the two groups, but patients in the G6PD^low^ group had significantly worse clinical outcomes, which seems to differ from our hypothesis. Besides, we explored the roles of other oxidative stress-related genes among SCLC patients. These patients with lower mRNA levels of HMOX1, NQO1, NFE2L2, and CAT had better survival (Fig. 6A), which was contradictory to the results of G6PD. Because of the interesting finding, we further investigated the prognostic role of G6PD in NSCLC patients to explore whether the prognostic role of G6PD was different between NSCLC and SCLC. GSE31210 including 246 NSCLC samples was selected for further analysis, and patients with lower mRNA levels of G6PD were significantly associated with longer OS and relapse-free survival (RFS) than those with higher expression (Fig. S6A, B), demonstrating the unique role of G6PD in SCLC.

In addition, we analyzed the RNA-seq datasets of primary radiosensitive and radioresistant SCLC cell lines from CCLE datasets [30], and found that most oxidative stress-related genes were upregulated in primary radioresistant cell lines other than G6PD (Fig. S6C). Similar results for most genes related to oxidative stress were found in our RNA-seq dataset between H446 and H446RR cells (Fig. S6D).

### PI3K/mTOR inhibitors overcome SCLC radioresistance by increasing ROS levels and reducing G6PD levels

To further validate whether PI3K/AKT/mTOR signaling contributed to SCLC radioresistance, insulin, the activator of this pathway, was combined with IR. Insulin effectively protected SBC2 and H446RR cells from specific cell death caused by IR (Fig. 6B), further suggesting that PI3K/AKT/mTOR pathway promoted the SCLC radioresistance.

To identify that ROS closely participated in the abrogation of SCLC radioresistance, N-acetylcysteine (NAC), the ROS scavenger, was combined with IR and BEZ235/GSK2126458. IR plus BEZ235/GSK2126458 markedly increased specific cell death in SBC2 and H446RR cells. However, NAC effectively reduced the specific cell death of SBC2 and H446RR cells triggered by the combination of IR and BEZ235/GSK216458 (Fig. 6C). The combination of NAC, IR and GSK216458 also significantly increased the clone number and clone size of SBC2 cells compared with cells treated with IR and GSK216458 (Fig. 6D), confirming that ROS was strongly involved in the abrogation of radioresistance.

6-aminonicotinamide (6-AN, the specific inhibitor of G6PD) was used to determine whether G6PD was involved with SCLC radioresistance. 6-AN had the synergetic effect of triggering specific cell death of SBC2 and H446RR cells when combined with IR and BEZ235/GSK216458 (Fig. 6E). 6-AN plus GSK2126458 plus IR notably reduced the clone number and clone size of SBC2 cells compared with cells treated with GSK2126458 plus IR (Fig. 6F). Moreover, the HBLV-ZsGreen-PURO and HBLV-G6PD-ZsGreen-3×flag-PURO lentiviruses were transfected into SBC2 cells. SCLC cells with G6PD overexpression were successfully constructed, as revealed by fluorescence microscopy and immunoblotting. The CCK8 assay showed that compared with cells in the mock-vehicle groups, cells transfected with the G6PD overexpression vector had higher cell proliferation rates when exposed to GSK2126458 alone, IR alone, or the combination of GSK2126458 and IR, suggesting that G6PD played a crucial role in SCLC radioresistance (Fig. 6G).

### PI3K/mTOR inhibitors promoted G6PD degradation through chaperone-mediated autophagy process to overcome SCLC radioresistance

**Fig. 7:**
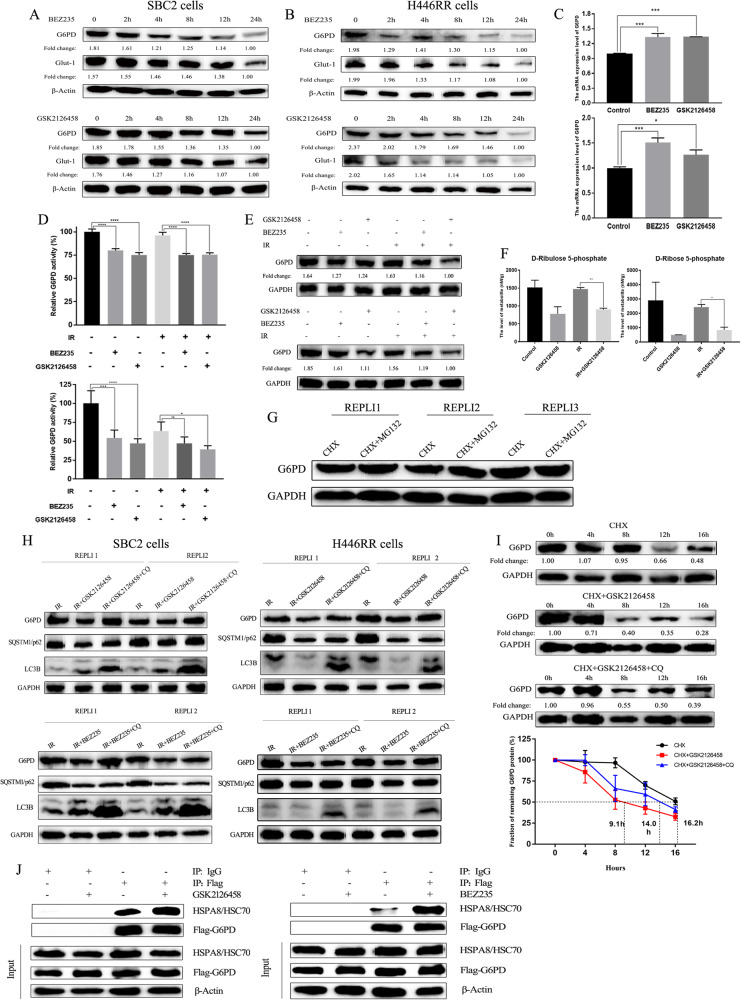
PI3K/mTOR inhibitors overcome the radioresistance of SCLC through increasing ROS production by promoting the autophagy-lysosome pathway of G6PD. **A**, **B** The protein expression of G6PD and Glut1 in SBC2 and H446RR cells treated with PI3K/mTOR inhibitors were detected by immunoblotting over time. **C** The mRNA level of G6PD and Glut1 in SBC2 and H446RR cells treated with PI3K/mTOR inhibitors were detected by q-PCR. **D** G6PD activity in SBC2 and H446RR cells treated with PI3K/mTOR inhibitors were detected. **E** G6PD expression in different groups was determined by immunoblotting. **F** Ribulose 5-phosphate and ribose 5-phosphate level were detected across different groups. **G** The expression of G6PD in cells treated with CHX (100 μg/ml for 6 h) or CHX + MG132 (10 μM for 6 h) were detected using immunoblotting. **H** The expression of G6PD, SQSTM1/p62 and LC3B II/I in cells treated with either IR alone, IR + PI3K/mTOR inhibitors, or IR + PI3K/mTOR inhibitors+CQ was detected by immunoblotting. **I** The half-life of G6PD protein in cells treated with either CHX alone, CHX + GSK2126458, or CHX + GSK2126458 + CQ was detected by immunoblotting. **J** The interaction between HSPA8/HSC70 and G6PD in SBC2 cells were validated by Co-IP assay.

BEZ235/GSK2126458 significantly reduced G6PD and Glut-1 protein levels in SBC2 and H446RR cells over time (Fig. 7A, B). However, G6PD mRNA expression was elevated in SBC2 cells treated with BEZ235/GSK2126458, and similar findings were reported in H446RR cells (Fig. 7C). These G6PD transcriptional and translational results were consistent with the finding between H446RR and H446 cells. G6PD activity was also downregulated in cells treated with BEZ235/GSK2126458 regardless of IR exposure (Fig. 7D). Subsequently, BEZ235/GSK2126458 effectively reduced G6PD protein expression with or without IR exposure (Fig. 7E). Compared with cells treated with IR alone, ribulose 5-phosphate and ribose 5-phosphate levels were significantly reduced in cells treated with GSK2126458 plus IR (Fig. 7F), which impeded DNA damage repair after exposure to IR.

Protein degradation typically occurs through two pathways: autophagic lysosomal pathway and proteasomal pathway. MG132 (an inhibitor of ubiquitin proteasome pathway) was utilized in our cell studies to investigate the potential role of the proteasomal pathway in G6PD degradation. Our findings indicated that the proteasomal pathway did not seem to be involved in G6PD degradation, as shown in Fig. 7G. To determine whether the autophagy-lysosome pathway participates in the regulation of G6PD under exposure to PI3K/mTOR inhibitors, chloroquine (CQ), an autophagy inhibitor, was combined with IR and PI3K/mTOR inhibitors. BEZ235/GSK2126458 could promote the degradation of G6PD protein mediated by the autophagy-lysosome pathway, with a reduction of SQSTM1/p62 and an increase of LC3B II/I ratio. Besides, reduced G6PD protein caused by BEZ235/GSK2126458 could be reversed by CQ, with an increase of SQSTM1/p62 and LC3B II/I ratio (Fig. 7H). Transmission electron microscopy (TEM) revealed that the number of autophagosomes was elevated in cells treated with GSK2126458, and the number of autophagolysosomes was increased in cells treated with GSK2126458 plus CQ under exposure to IR (Fig. S7A).

Compared to cells treated with only CHX, GSK2126458 considerably shortened the half-life of G6PD protein, but the reduction in the G6PD half-life was partially blocked by CQ (Fig. 7I). Furthermore, Co-IP assay was carried out in cells with G6PD overexpression to determine the type of G6PD autophagic degradation and the autophagy-associated molecule which interacted with G6PD. Mass spectrometric analysis showed that HSPA8/HSC70 was found in G6PD-associated proteins (Table S5). Co-IP assay further confirmed the interaction between G6PD and HSPA8/HSC70, and this interaction could be reinforced by GSK2126458 (Fig. 7J). These findings indicated that PI3K/mTOR inhibitors accelerated G6PD degradation through chaperone-mediated autophagy process.

### PI3K/mTOR inhibitors overcome the radioresistance of SCLC in vivo

**Fig. 8:**
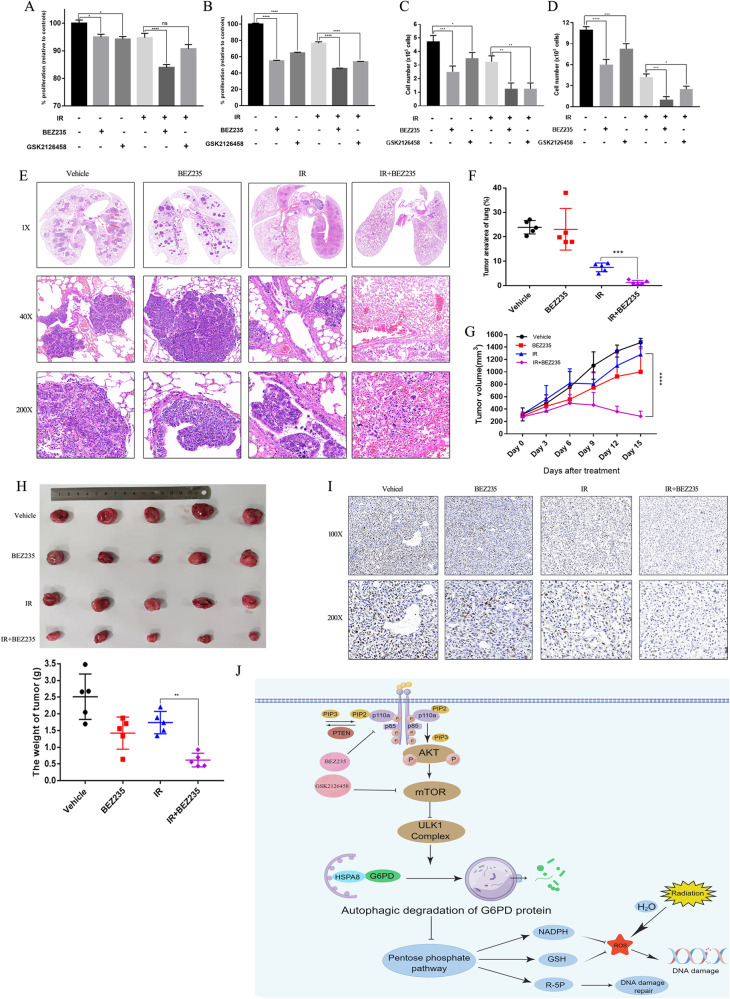
PI3K/mTOR inhibitors overcome the radioresistance of SCLC in vivo. **A**–**D** The cell proliferation of two murine SCLC cell lines (TKO-tdTomato and TKO-mTmG) treated with different treatments were determined by CCK8 assay. **E** The HE staining of the whole lung in TKO mice from various groups was performed. **F** The tumor area/area of the lung in TKO mice from various groups was quantitatively calculated. **G**, **H** The volume and weight of allograft tumors in TKO mice treated with PI3K/mTOR inhibitors combined with or without IR was detected. **I** The expression of Ki67 in allograft tumor samples from TKO mice in different groups were determined by IHC. **J** PI3K/AKT/mTOR pathway promoted SCLC radioresistance by regulating the pentose phosphate pathway, and PI3K/mTOR inhibitors overcome SCLC radioresistance through increasing ROS level and reducing G6PD expression. PI3K/mTOR inhibitors promoted the interaction between G6PD and HSPA8/HSC70, and accelerated G6PD degradation through chaperone-mediated autophagy process.

The mouse SCLC model, Cgrp^CreER^;TKO, was used to determine whether PI3K/mTOR inhibitors could overcome the SCLC radioresistance. First, we tested the cytotoxicity of BEZ235 and GSK2126458 in two mouse SCLC cells acquired from Cgrp^CreER^;TKO mice. BEZ235 plus IR had better anti-cancer activity than the combination of GSK2126458 and IR (Fig. 8A-D and Fig. S7B-E). Therefore, BEZ235 was selected for further mouse experiments. Hematoxylin-eosin (HE) staining of the lung suggested that mice treated with BEZ235 combined with IR had significantly increased tumor regression compared with mice treated with BEZ235 alone or IR alone (Fig. 8E). Quantitative analysis showed that tumor size and number was significantly reduced in mice treated with BEZ235 and IR than mice treated with either modality alone, further suggesting that inhibiting PI3K/AKT/mTOR pathway sensitized SCLC cells to radiation in vivo (Fig. 8F). In addition, HE staining results showed that no apparent toxicity of the heart, liver, and kidney was observed in mice treated with BEZ235 and IR, suggesting the safety of this combination (Fig. S7F).

To further determine the anti-cancer activity of the combination of IR and BEZ235 in vivo, allograft transplantation assay was performed using murine SCLC cells with tdTomato. Compared with mice treated with either modality alone, tumor volume and weight were reduced in mice treated with IR plus BEZ235 (Fig. 8G, H), revealing that the combination of BEZ235 and IR could effectively inhibit the allograft tumor growth in vivo. Additionally, the combination of BEZ235 and IR inhibited cell proliferation as revealed by Ki67 detection in allograft tumor samples (Fig. 8I).

## Discussion

Our study further validated our previous investigation of PI3K/AKT signaling in SCLC therapeutic resistance [7] and revealed the connection between PI3K/AKT/mTOR signaling and the PPP underlying SCLC radioresistance. PI3K/AKT/mTOR pathway contributes to the development of primary and acquired radioresistance in SCLC cells. Pharmacological inhibition of PI3K/AKT/mTOR signaling rewired redox homeostasis and intensified oxidative stress damage (the increased ROS accumulation and reduced NAPDH levels) by inhibiting G6PD expression and activity, resulting in enhanced apoptosis in cells treated with PI3K/mTOR inhibitors plus IR. In detail, PI3K/mTOR inhibitors promoted the interaction between G6PD and HSPA8/HSC70, and accelerated G6PD degradation through chaperone-mediated autophagy process (Fig. 8J). GEMM and allograft transplantation assays further confirmed that PI3K/mTOR inhibitors could effectively augment anti-tumor efficacy of IR.

SCLC is highly sensitive to radiotherapy in the early stages of treatment, but radioresistance inevitably develops in a significant proportion of patients [32, 33]. Cancer radioresistance is an intricate phenomenon that has attracted a lot of attention, and there are two main hypotheses to explain it [34, 35]. First, tumor cells with both high and low sensitivity to radiation exist in tumors. Under radiation exposure, tumor cells highly sensitive to radiation were severely inhibited, while cells those with low sensitivity could survive, rapidly proliferate, and eventually achieve a dominant position, which is known as intrinsic radioresistance. Second, most cancer cells are killed by radiation, but a few individual cells selected by multiple fractionated radiotherapy gradually undergo adaptation and evolution, known as acquired radioresistance. According to their radiosensitivity in multiple SCLC cell lines, SBC2 cells were used as the primary radioresistant cell model in our study, while H446 cells were used to develop acquired radioresistance by irradiating H446 cells with fractionated doses of X-rays. Compared with other studies related to SCLC radioresistance, our study had some obvious advantages. Li, et al. also found dual histone deacetylase (HDAC) and PI3K inhibitor, FK228, was reported to overcome SCLC radioresistance in vitro. But only primary radioresistant SCLC cells were used in their analysis, instead of establishing acquired radioresistant SCLC cells. Moreover, only cell experiments were performed in their study without any animal experiments, and animal experiments were very significant to explore the therapeutic efficacy of specific inhibitors combined with IR [28, 36]. First, to our knowledge, H446RR cells successfully established in this study were the first SCLC cells with acquired radioresistance. Second, the differences between H446RR cells and their parental cells were first compared using multiple approaches, including RNA-seq analysis and targeted metabolomics. Third, Cgrp^CreER^;TKO mice were first used to investigate efficacy and side effects of treatment in our study, and their tumors existed in the lungs instead of subcutaneous tissue, which better mimicked the clinical setting of SCLC patients compared with subcutaneous transplantation models.

PI3K/AKT/mTOR signaling is associated with the enhanced proliferation and growth of cancer cells, and its activation was strongly correlated with unfavorable survival and resistance to radiotherapy [37, 38]. Comprehensive genomic analyses have identified that 7–36% of SCLC harbor *PTEN, PIK3CA, AKT2, AKT3, RICTOR*, and *MTOR* mutations, suggesting the frequent dysregulation of PI3K/AKT/mTOR pathway in SCLC [39, 40]. Recently, one study revealed that PI3K/AKT/mTOR signaling promoted the phenotypic transition from suspension to adhesion growth patterns and conferred SCLC cells with chemo-resistance [27]. Our previous analysis comparing paired SCLC samples at diagnosis and relapse identified that genes belonging to PI3K/AKT pathway were significantly enriched [7]. IR could result in p53-dependent or independent G1 and G2 cell cycle arrest, but PI3K/AKT/mTOR signaling could override p53-independent cell cycle arrest through the activation of cyclin D and the inactivation of cell cycle-dependent kinase inhibitor p27, ultimately promoting cell proliferation and radioresistance [41]. Three crucial kinases, DNA-dependent protein kinase catalytic subunit (DNA-PKcs), ataxia-telangiectasia mutated (ATM), and ATM and RAD3-related proteins (ATR) were closely involved in DNA damage repair [42–44]. Consistent with our findings, BEZ235 was found to potently suppress ATM and DNA-PKcs kinases, attenuate DNA damage repair, and augment therapeutic effect of radiotherapy in glioblastoma (GBM) [36, 37]. Although the effects of PI3K/AKT/mTOR signaling on these key kinases in DNA damage repair have been frequently reported, the crosstalk between PI3K/AKT/mTOR pathway, the PPP and radioresistance were not explored.

It is widely recognized that radiation can increase tyrosine phosphorylation of EGFR signaling, and its phosphorylation promotes postirradiation repopulation of cancer cells [45, 46]. EGFR signaling also induces the downstream activation of HIF-1 and PI3K/AKT/mTOR signaling, which rewires glycolysis and the PPP [47]. The PI3K/AKT/mTOR pathway not only increased glycolysis by elevating the expression of glucose transporters and the glycolytic rate but also decoupled glycolysis and the TCA cycle and diverted metabolic intermediates to the PPP branching metabolic pathway [48, 49]. The increased flux in glycolysis and the PPP provided survival benefits to cancer cells after radiation. As the key enzyme of the PPP, G6PD primarily regulates its oxidative branch and determines the speed of glycolytic intermediate flux into the PPP. The oxidative branch of the PPP is an irreversible stage and plays a significant role in providing ribonucleotides and NADPH to generate nucleotides and reduced glutathione (GSH), both of which are beneficial for the development of radioresistance [50]. Rapidly dividing tumor cells usually upregulate this pathway when exposed to radiation-induced oxidative stress and DNA damage. Our results showed that 6-AN, the specific inhibitor of G6PD, could improve the antitumor effect of IR in SCLC cells. Similarly, G6PD-deficient CHO cells showed significantly higher sensitivity to IR than wild-type cells when exposed to single-dose or fractionated radiation, and the radiosensitivity disappeared when the cDNA for wild-type G6PD was transfected [51]. These outcomes demonstrated that G6PD inhibition was an effective strategy to abrogate radioresistance.

However, the connection between PI3K/AKT/mTOR signaling and the PPP has not been well understood until now. The correlation between mTOR signaling and the PPP was explored in mouse embryonic fibroblasts using gene set enrichment analysis (GSEA), and genes related to the PPP were identified to be mTORC1-induced genes in *Tsc1*^−/−^ and *Tsc2*^−/−^ cells. mTORC1 was found to increase G6PD mRNA expression by activating HIF1α- and SREBP-mediated transcription [52]. The inhibition of *TSC2* was found to activate mTORC1 signaling in GBM cells, and G6PD was considerably upregulated in cells transfected with the pLKO.1 plasmids targeting *TSC2* compared to cells transfected with the pLKO.1 plasmid with a non-targeting shRNA sequence. Torin2 and rapamycin, mTOR inhibitors, reduce G6PD expression to varying degrees [53]. Androgen receptor signaling was reported to promote the PPP through mTOR-mediated upregulation of G6PD in human prostate cancer cell lines, and G6PD expression was closely correlated with prostate cancer progression using two animal models of Pten deletion/elevated mTOR pathway [54]. A recent study showed that PI3K/AKT activation increased the half-life of the G6PD protein through the inhibition of TRIM21-mediated ubiquitination, and the TRIM21 level had a negative correlation with PI3K/AKT activity in many human cancers [21]. However, our study revealed a slight disparity as TRIM21 was not detected among the G6PD-associated proteins identified through mass spectrometric analysis. Furthermore, our findings from the MG132 experiment indicated that the proteasomal pathway may not be implicated in G6PD degradation in SCLC. Instead, chaperone-mediated autophagy was involved in G6PD degradation. The inconsistence might be caused by different cancer cell lines. Moreover, the mTOR inhibitor, everolimus, was reported to abrogate resistance to dexamethasone in T-cell acute lymphoblastic leukemia cells by reducing G6PD protein levels and enhancing ROS levels [55]. Our study has elaborated that PI3K/mTOR inhibitors reinforced the interaction between HSPA8/HSC70 and G6PD, accelerated G6PD degradation and reduced G6PD protein level. Further studies were necessary to characterize the specified molecular mechanism of G6PD degradation in various cancers.

## Conclusion

PI3K/mTOR signaling pathway plays a critical role in the development of primary and acquired resistance to radiation in SCLC cells. PI3K/mTOR inhibitors were a feasible therapeutic approach to sensitize SCLC cells to radiation by promoting G6PD autophagic degradation and aggravating oxidative stress damage, with no apparent side effects.

### Supplementary information

Orignial data of WB

Supplementary legends

Table S1

Table S2

Table S3

Table S4

Table S5

Figure S1

Figure S2

Figure S3

Figure S4

Figure S5

Figure S6

Figure S7

## Data Availability

We have deposited our RNA-seq datasets on the following repository: https://figshare.com/s/b2f4f20d1a3173308b0b.
