# Supplementary material for: PI3K/mTOR inhibitors promote G6PD autophagic degradation and exacerbate oxidative stress damage to radiosensitize small cell lung cancer
Source: Cell Death Dis. 2023 Oct 6;14(10):652. doi: 10.1038/s41419-023-06171-7 (PMC10558571; doi:10.1038/s41419-023-06171-7)
Supplement: Supplementary file 2 — Supplementary legends [file 41419_2023_6171_MOESM2_ESM.docx]

**Supplementary materials**

**Figure S1** Gene ontology (GO) and kyoto encyclopedia of genes and genomes (KEGG) pathway analyses in lung cancer cells 24 h after10 Gy x-ray radiation.

**Figure S2** The correlation between PI3K/AKT/mTOR pathway upregulation after ionizing radiation and the primary radioresistance.

**Figure S3** The effect of PI3K/mTOR inhibitors in SCLC cells.

**Figure S4** PI3K/mTOR inhibitors increase the cytotoxic effects and DNA damage caused by IR in H446 and DMS53 cells.

**Figure S5** The difference between H446RR cells and their parental H446 cells.

**Figure S6** Bioinformatic analysis of G6PD role in radioresistant cells.

**Figure S7** BEZ235 overcomes the radioresistance of SCLC *in vivo*.

**Supplementary table S1** The detailed information of antibody

**Supplementary table S2** The primers of genes

**Supplementary table S3** Differential gene expression in RNA-seq analysis

**Supplementary table S4** The difference in glucose metabolism between H446-RR and H446 cells using targeted metabolomics

**Supplementary table S5** G6PD-associated proteins revealed by mass spectrometric analysis

**Figures legend**

**Figure S1** Gene ontology (GO) and kyoto encyclopedia of genes and genomes (KEGG) pathway analyses in lung cancer cells treated with or without x-ray radiation. **A-B** GO and KEGG pathway analysis in SBC2 cells 24 h after 10 Gy x-ray radiation. **C-D** GO and KEGG pathway analysis in A549 cells 24 h after 10 Gy x-ray radiation. **E-F** GO and KEGG pathway analysis in H446 cells 24 h after 10 Gy x-ray radiation.

**Figure S2** The correlation between PI3K/AKT/mTOR pathway upregulation after IR and the primary radioresistance. **A-C** The upregulation of PI3K/AKT signaling was observed 24 h after IR in SBC2, H446, and DMS53 cells. **D** Proteins related to PI3K/AKT pathway after IR in SBC2, H446 and DMS53 cells were quantitatively analyzed. **E** Growth inhibition of SBC2, H446 and DMS53 cells were determined by CCK8 assay at 72 h after 6 Gy of X-ray irradiation. **F-G** Glucose intake and lactic acid production in SBC2 cells treated with PI3K/mTOR inhibitors were detected. **H-I** The cell proliferation of in SBC2 cells were determined by CCK8 assay 24 h, 48 h, and 72 h after exposure to PI3K/mTOR inhibitors.

**Figure S3** The effect of PI3K/mTOR inhibitors in SCLC cells. **A-B** The impact of PI3K/mTOR inhibitors on glucose intake and lactic acid production in H466 and DMS53 cells, respectively. **C-D** The cell proliferation of in H446 and DMS53 cells were determined by CCK8 assay 24 h, 48 h, and 72 h after exposure to PI3K/mTOR inhibitors. **E-F** The key proteins involved in the PI3K/AKT/mTOR signaling pathway in H446 and DMS53 cells were detected 2 h after radiotherapy. **G-H** The proliferation of H446 and DMS53 cells using different treatments were detected 72 h after radiotherapy.

**Figure S4** PI3K/mTOR inhibitors increase the cytotoxic effects of IR in H446 and DMS53 cells. **A-B** The cell apoptosis of DMS53 cells using different treatment were determined by Annexin V/PI stain. **C-D** The expression of PARP and cleaved-PARP in H446 and DMS53 cells were determined by immunoblotting. **E-H** The expression of γ-H2AX in H446 and DMS53 cells were determined by immunofluorescence and immunoblotting. **I** The expression of p-ATM and p-CHK2 in SBC2 cells across different groups were detected using immunoblotting 45 and 90 min after radiotherapy.

**Figure S5** The difference between H446RR and its parental H446 cells. **A** The differential expression analysis and the KEGG pathway analysis in U87MG cells and their radioresistant cell lines in GSE207002. **B** The differential expression analysis and the KEGG pathway analysis in MCF-7 cells and their acquired radioresistant cells in GSE210411. **C-F** The important metabolites of energy metabolism which mainly included glycolysis, PPP, and TAC cycle were obtained by quantitative metabolomics, and the differences of metabolites between H446-RR and H446 cells were compared.

**Figure S6** Bioinformatic analysis revealed the roles of oxidative stress-related genes in SCLC radioresistance. **A-B** The correlation between G6PD and survival (overall survival (OS) and recurrence-free survival (RFS)) among NSCLC patients. **C** The expression of oxidative stress-related genes in the primary radioresistant and radiosensitive cells according to the dataset of SCLC cell lines from the Cancer Cell Line Encyclopedia (CCLE) platform. **D** The expression of oxidative stress-related genes between H446 and H446RR in our RNA-seq dataset. **E** The half-life of G6PD protein in cells treated with either CHX alone, CHX+BEZ235, or CHX+BEZ235+CQ was detected by immunoblotting.

**Figure S7** BEZ235 overcomes the radioresistance of SCLC *in vivo*. **A** Transmission electron microscope (TEM) revealed the number of autophagosomes and autophagolysosomes in cells treated with GSK2126458, or GSK2126458 + CQ under exposure to IR. **B-E** Bright field image and intensity of fluorescence in two murine SCLC cells (TKO-Tdtomato and TKO-mTmG cells) treated with PI3K/mTOR inhibitors combined with or without IR. **F** HE staining results of the heart, liver, and kidney from TKO mice treated with different therapies to evaluate potential side effects.
