# Supplementary material for: PI3K/mTOR inhibitors promote G6PD autophagic degradation and exacerbate oxidative stress damage to radiosensitize small cell lung cancer
Source: Cell Death Dis. 2023 Oct 6;14(10):652. doi: 10.1038/s41419-023-06171-7 (PMC10558571; doi:10.1038/s41419-023-06171-7)
Supplement: Supplementary file 3 — Table S1 [file 41419_2023_6171_MOESM3_ESM.docx]

| **Antibody** | **Manufacturer** | **Catalog** | **Dilution** | **Assay** |
| --- | --- | --- | --- | --- |
| anti-rabbit Akt | Cell signaling Technology  (TX, USA) | 4691S | 1:1000 | WB |
| anti-rabbit Phospho-Akt | Cell signaling Technology  (TX, USA) | 4060S | 1:1000 | WB |
| anti-rabbit Phospho-Akt | Cell signaling Technology  (TX, USA) | 13038T | 1:1000 | WB |
| anti-rabbit 4E-BP1 | Cell signaling Technology  (TX, USA) | 9644S | 1:1000 | WB |
| anti-rabbit Phospho-4E-BP1 | Cell signaling Technology  (TX, USA) | 4060S | 1:1000 | WB |
| anti-rabbit p70 S6 Kinase | Cell signaling Technology  (TX, USA) | 2708S | 1:1000 | WB |
| anti-rabbit Phospho-p70 S6 Kinase | Cell signaling Technology  (TX, USA) | 9205S | 1:1000 | WB |
| anti-rabbit mTOR | Cell signaling Technology  (TX, USA) | 2983S | 1:1000 | WB |
| anti-rabbit Phospho-mTOR | Cell signaling Technology  (TX, USA) | 5536S | 1:1000 | WB |
| anti-rabbit Phospho-Histone H2A.X | Cell signaling Technology  (TX, USA) | 2577S | 1:1000  1:400 | WB  IF |
| anti-rabbit Cleaved PARP | Cell signaling Technology  (TX, USA) | 5625S | 1:1000 | WB |
| anti-rabbit PARP | Cell signaling Technology  (TX, USA) | 9532S | 1:1000  1:400 | WB  IF |
| anti-rabbit G6PD | Abcam Technology (MA, USA) | ab210702 | 1:1000 | WB |
| anti-phospho-ATM | Abcam Technology (MA, USA) | ab81292 | 1:3000 | WB |
| anti-rabbit Glut | Abcam Technology (MA, USA) | ab115730 | 1:1000 | WB |
| anti-rabbit SQSTM1 / p62 | Abcam Technology (MA, USA) | ab109012 | 1:2000 | WB |
| anti-rabbit LC3B | Abcam Technology (MA, USA) | ab192890 | 1:2000 | WB |
| anti‐rabbit Phospho Nrf2 | Abcam Technology (MA, USA) | ab76026 | 1:1000 | WB |
| anti‐rabbit Ki67 | Abcam Technology (MA, USA) | ab16667 | 1:200 | IHC |
| anti‐mouse Flag | Proteintech Group  (Shanghai, China) | 66008-4-g | 1:5000 | WB |
| anti‐rabbit Phospho-CHEK2 | Proteintech Group  (Shanghai, China) | 29012-1-AP | 1:1000 | WB |
| anti‐mouse Beta Actin | Proteintech Group  (Shanghai, China) | 66009-1-Ig | 1:5000 | WB |
| anti‐mouse GAPDH | Proteintech Group  (Shanghai, China) | 66004-1-Ig | 1:5000 | WB |

**Supplementary Table S1** **The detailed information of antibody**
