# Supplementary material for: PI3K/mTOR inhibitors promote G6PD autophagic degradation and exacerbate oxidative stress damage to radiosensitize small cell lung cancer
Source: Cell Death Dis. 2023 Oct 6;14(10):652. doi: 10.1038/s41419-023-06171-7 (PMC10558571; doi:10.1038/s41419-023-06171-7)
Supplement: Supplementary file 4 — Table S2 [file 41419_2023_6171_MOESM4_ESM.docx]

| **Supplementary table S2 The primers of genes** | | |
| --- | --- | --- |
| **Gene** | **The forward primer** | **The reverse primer** |
| G6PD | CCGCAAACAGAGTGAGCCCTTC | AGGACTCGTGAATGTTCTTGGTGAC |
| GLUT1 | AAACATGGAACCACCGCTAC | AACAAAGAGGCCGACAGAGA |
| GAPDH | GGAGCGAGATCCCTCCAAAAT | GGCTGTTGTCATACTTCTCATGG |
