# Supplementary material for: PI3K/mTOR inhibitors promote G6PD autophagic degradation and exacerbate oxidative stress damage to radiosensitize small cell lung cancer
Source: Cell Death Dis. 2023 Oct 6;14(10):652. doi: 10.1038/s41419-023-06171-7 (PMC10558571; doi:10.1038/s41419-023-06171-7)
Supplement: Supplementary file 7 — Table S5 [file 41419_2023_6171_MOESM7_ESM.docx]

**Table S5 G6PD-associated proteins revealed by mass spectrometric analysis**

| **Accession** | **Protein names** | **Gene names** | **MW [kDa]** | **Protein score** | **Sequence coverage (%)** | **Unique Peptides** | **Peptides** | **PSMs** |
| --- | --- | --- | --- | --- | --- | --- | --- | --- |
| P31943 | Heterogeneous nuclear ribonucleoprotein H | HNRNPH1 | 49.2 | 2236 | 54 | 11 | 19 | 40 |
| P35579 | Myosin-9 | MYH9 | 226.4 | 1560 | 21 | 24 | 32 | 37 |
| P52597 | Heterogeneous nuclear ribonucleoprotein F | HNRNPF | 45.6 | 1430 | 47 | 10 | 13 | 27 |
| P55795 | Heterogeneous nuclear ribonucleoprotein H2 | HNRNPH2 | 49.2 | 1427 | 41 | 8 | 15 | 31 |
| P11940 | Polyadenylate-binding protein 1 | PABPC1 | 70.6 | 1362 | 43 | 13 | 19 | 29 |
| Q9Y2W1 | Thyroid hormone receptor-associated protein 3 | THRAP3 | 108.6 | 1269 | 25 | 21 | 23 | 33 |
| Q13310 | Polyadenylate-binding protein 4 | PABPC4 | 70.7 | 1192 | 34 | 12 | 18 | 26 |
| P61978 | Heterogeneous nuclear ribonucleoprotein K | HNRNPK | 50.9 | 1148 | 48 | 17 | 17 | 25 |
| P09651 | Heterogeneous nuclear ribonucleoprotein A1 | HNRNPA1 | 38.7 | 1133 | 46 | 13 | 16 | 24 |
| Q96PK6 | RNA-binding protein 14 | RBM14 | 69.4 | 1020 | 31 | 17 | 17 | 21 |
| P11142 | Heat shock cognate 71 kDa protein | HSPA8 | 70.9 | 1008 | 30 | 13 | 16 | 21 |
| P08670 | Vimentin | VIM | 53.6 | 673 | 35 | 14 | 16 | 17 |
| Q14134 | Tripartite motif-containing protein 29 | TRIM29 | 65.8 | 670 | 29 | 15 | 15 | 19 |

Abbreviation: PSM, Number of peptide-spectrum matches.
