# Supplementary figures and images for: PI3K/mTOR inhibitors promote G6PD autophagic degradation and exacerbate oxidative stress damage to radiosensitize small cell lung cancer
Source: Cell Death Dis. 2023 Oct 6;14(10):652. doi: 10.1038/s41419-023-06171-7 (PMC10558571; doi:10.1038/s41419-023-06171-7)

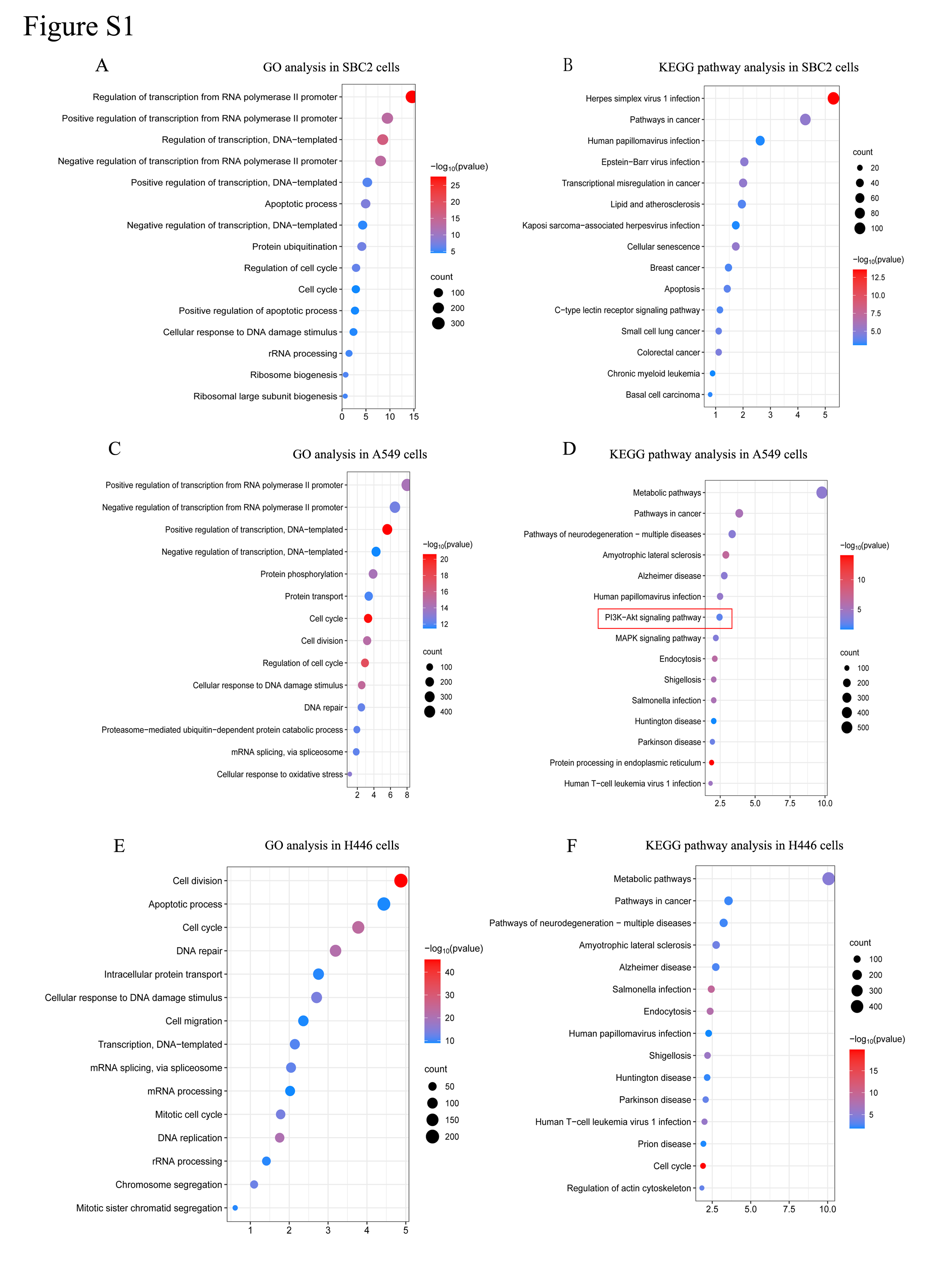

Supplement: Supplementary file 8 — Figure S1 [file 41419_2023_6171_MOESM8_ESM.tif]

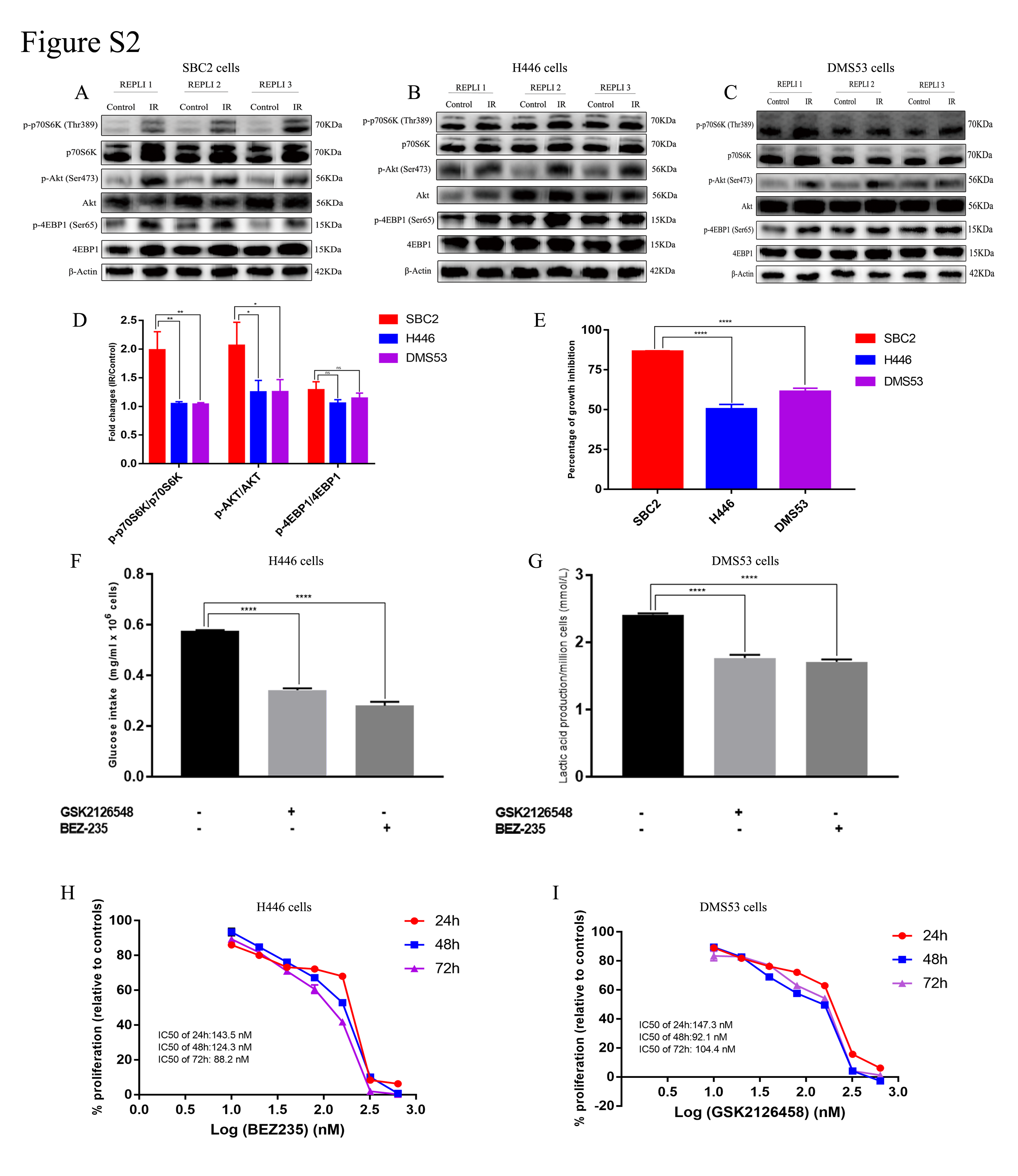

Supplement: Supplementary file 9 — Figure S2 [file 41419_2023_6171_MOESM9_ESM.tif]

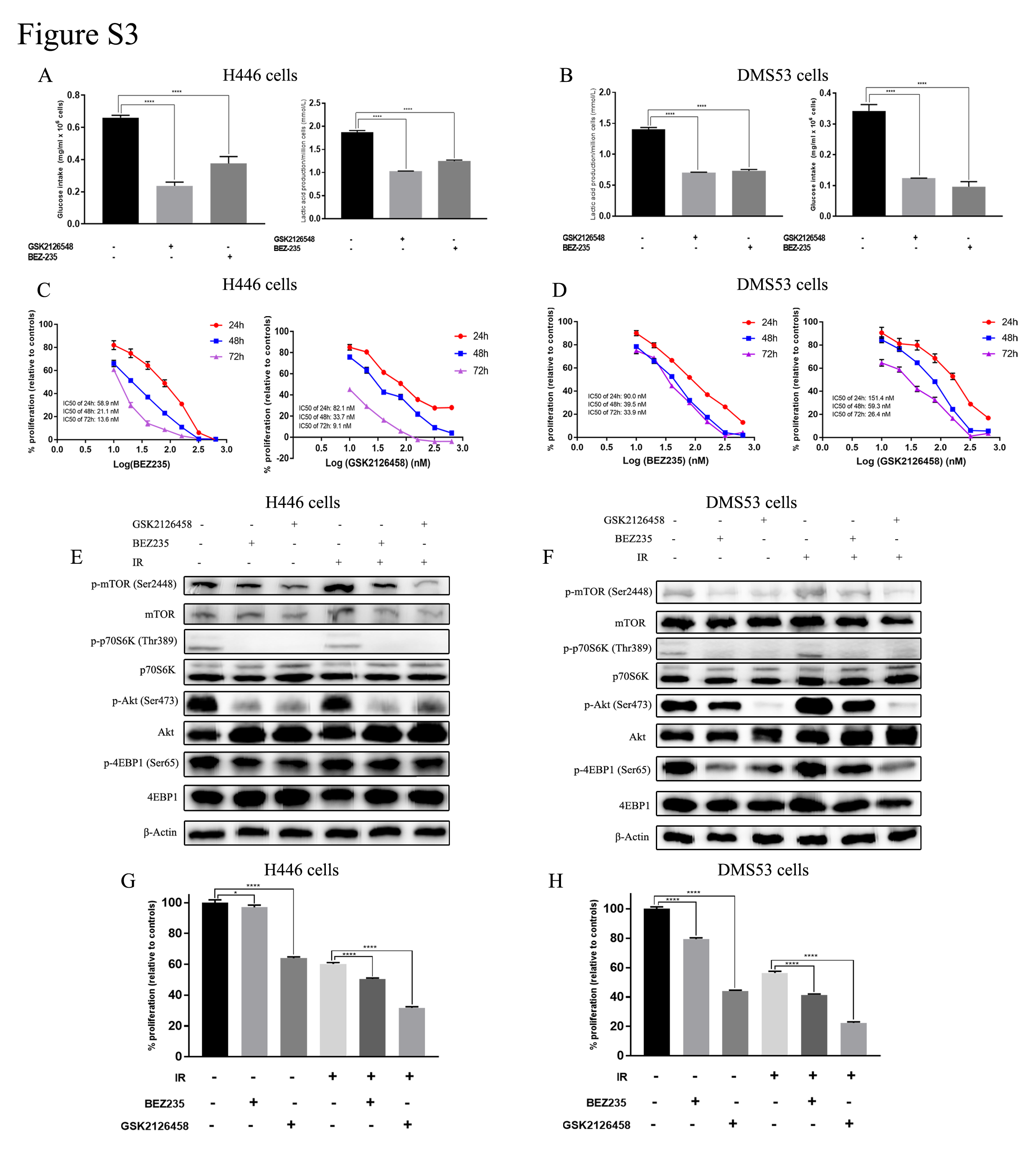

Supplement: Supplementary file 10 — Figure S3 [file 41419_2023_6171_MOESM10_ESM.tif]

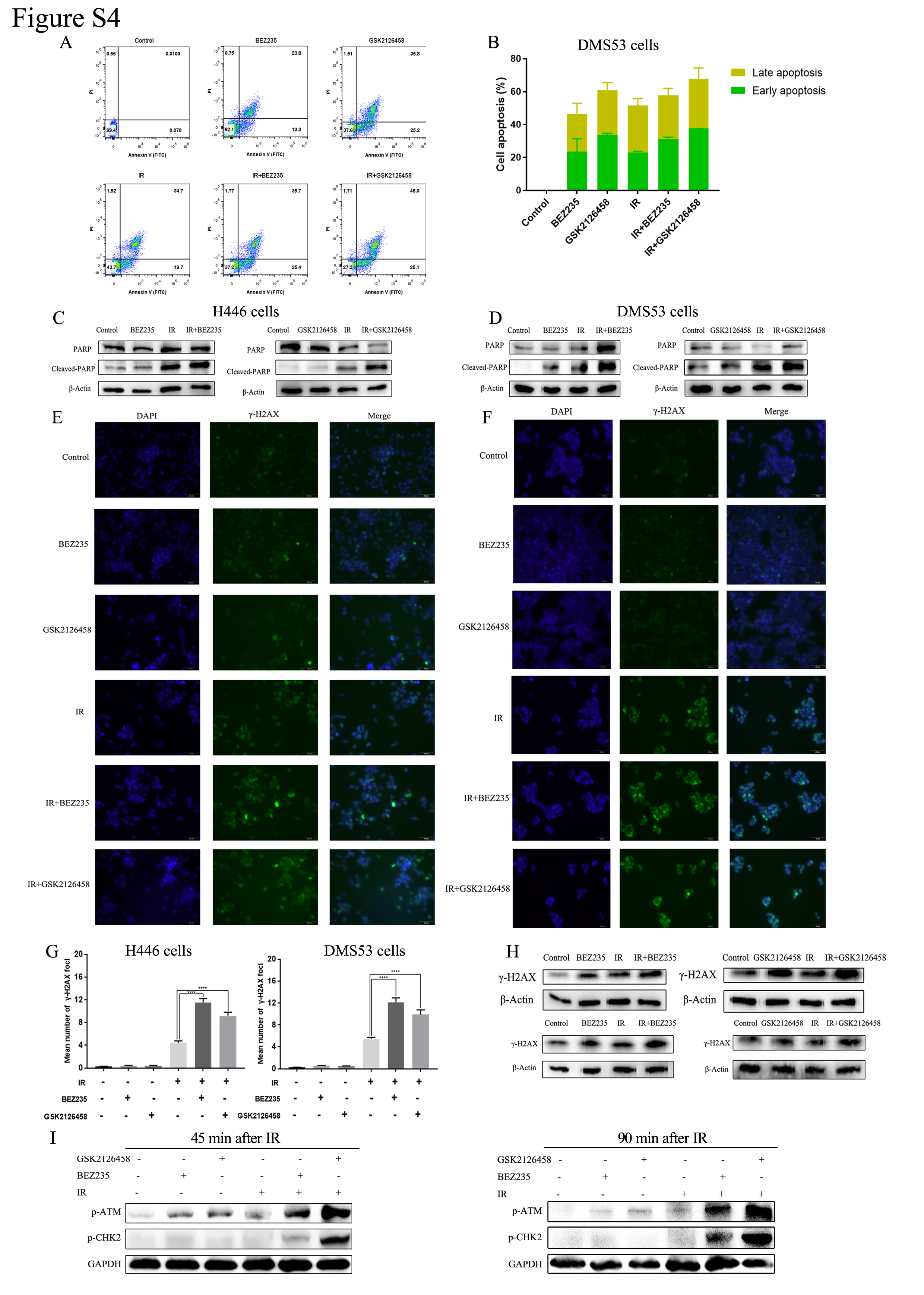

Supplement: Supplementary file 11 — Figure S4 [file 41419_2023_6171_MOESM11_ESM.tif]

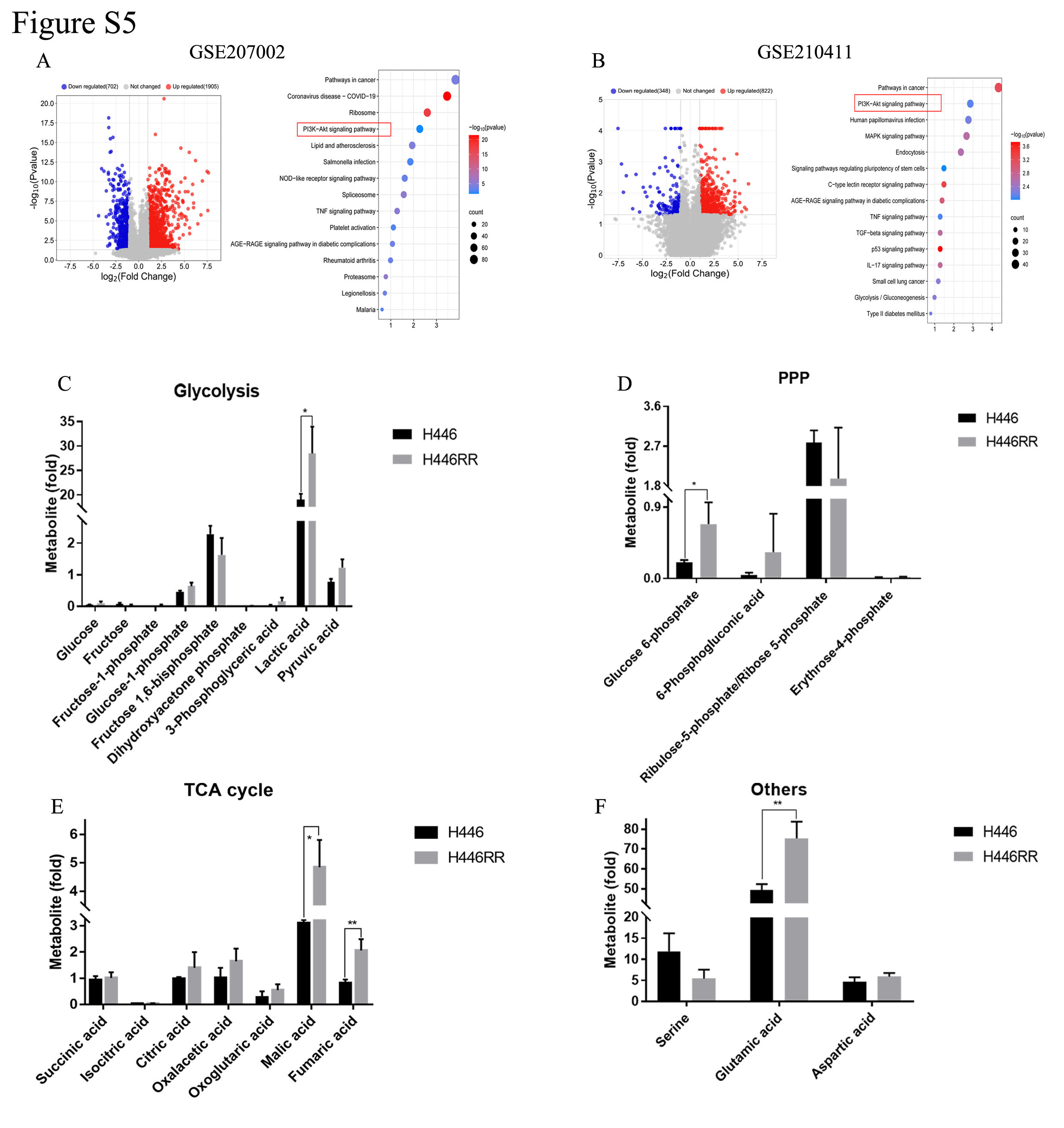

Supplement: Supplementary file 12 — Figure S5 [file 41419_2023_6171_MOESM12_ESM.tif]

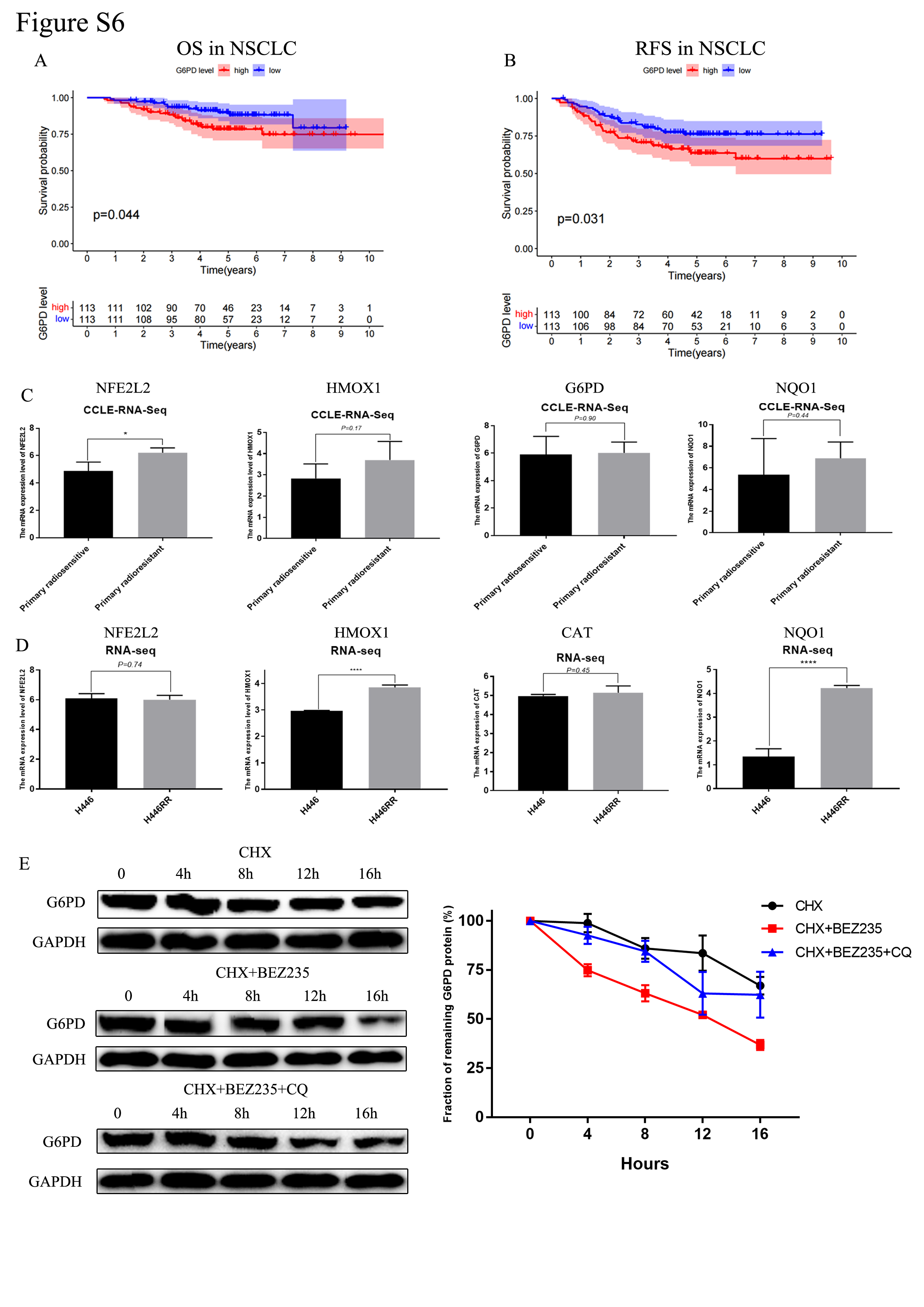

Supplement: Supplementary file 13 — Figure S6 [file 41419_2023_6171_MOESM13_ESM.tif]

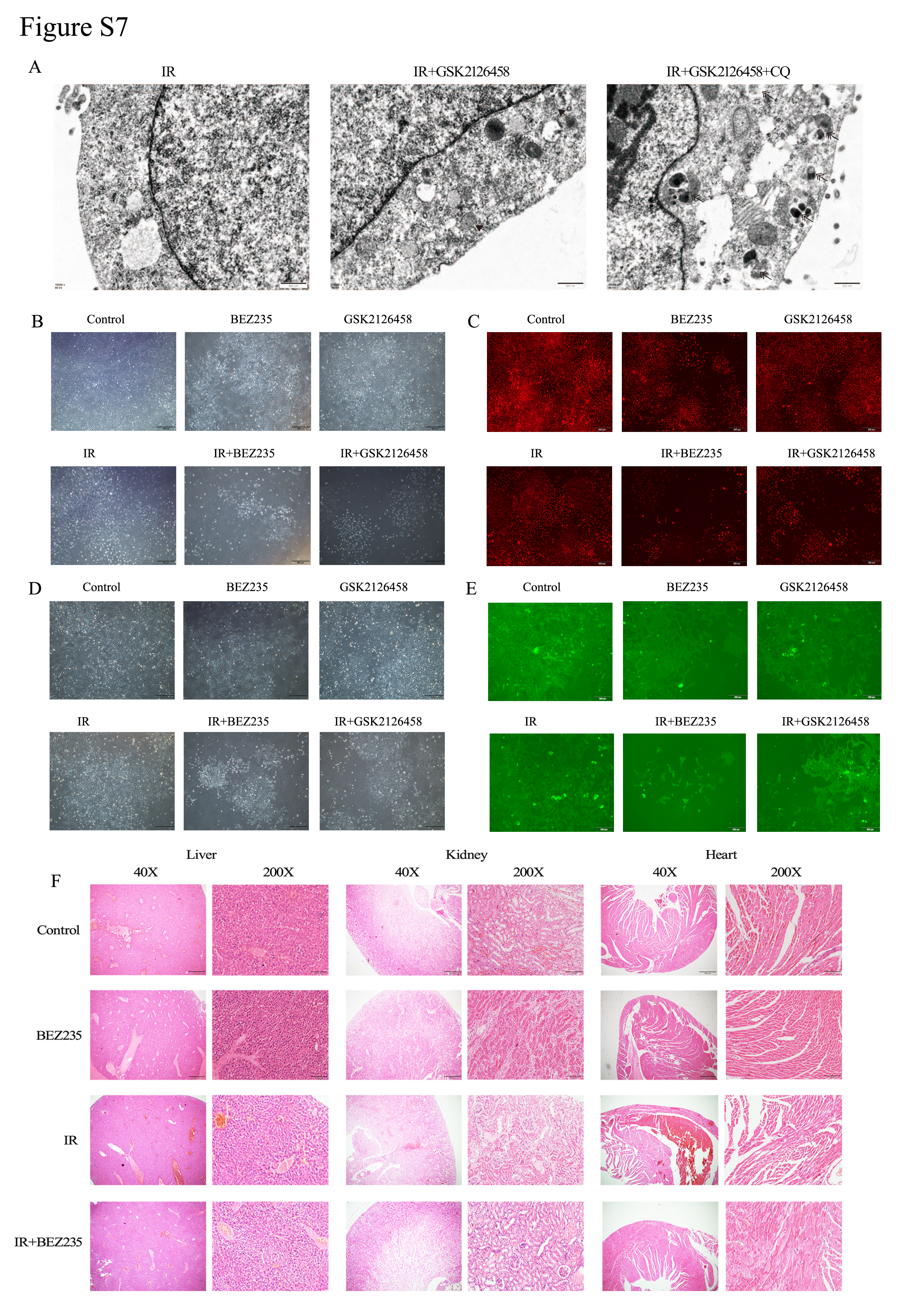

Supplement: Supplementary file 14 — Figure S7 [file 41419_2023_6171_MOESM14_ESM.tif]
