# Supplementary material for: PI3K/mTOR inhibitors promote G6PD autophagic degradation and exacerbate oxidative stress damage to radiosensitize small cell lung cancer
Source: Cell Death Dis. 2023 Oct 6;14(10):652. doi: 10.1038/s41419-023-06171-7 (PMC10558571; doi:10.1038/s41419-023-06171-7)
Supplement: Supplementary file 1 — Orignial data of WB [file 41419_2023_6171_MOESM1_ESM.docx]

**Figure 1A**

p-mTOR (Ser2448)

mTOR

p-4EBP1(Ser65)

4EBP1

p-70S6K (Thr389)

p70S6K

p-AKT (Ser473)

AKT

p-AKT (Thr308)

AKT

β-Actin

**Figure 1G**

Cleaved PARP

PARP

β-Actin

Cleaved PARP

PARP

β-Actin

Cleaved PARP

PARP

β-Actin

Cleaved PARP

PARP

β-Actin

**Figure 2C**

γ-H2AX

β-Actin

γ-H2AX

β-Actin

**Figure 3B**

Cleaved-PARP

PARP

β-Actin

**Figure 3C**

p-mTOR (Ser2448)

mTOR

p-p70S6K (Thr389)
p70S6K

p-AKT (Ser473)

AKT

p-4EBP1 (Ser65)

4EBP1

β-Actin

**Figure 3D**

Glut1

β-Actin

G6PD

β-Actin

**Figure 4B**

PARP

Cleaved-PARP

β-Actin

**Figure 5C**

γ-H2AX

β-Actin

**Figure 6G**

G 6PD-ZsGreen

G6PD

GAPDH

**Figure 7A**

Glut-1

G6PD

β-Actin

Glut-1

G6PD

β-Actin

**Figure 7B**

Glut-1

G6PD

β-Actin

Glut-1

G6PD

β-Actin

**Figure 7E**

G6PD

GAPDH

G6PD

GAPDH

**Figure 7G**

G6PD

GAPDH

**Figure 7H**

G6PD

P62/SQSTM1

LC3B

GAPDH

G6PD

P62/SQSTM1

LC3B

GAPDH

G6PD

P62/SQSTM1

LC3B

GAPDH

G6PD

P62/SQSTM1

LC3B

GAPDH

**Figure 7I**

G6PD

GAPDH

G6PD

GAPDH

G6PD

GAPDH

**Figure 7J**

HSPA8/HSC70

Flag-G6PD

HSPA8/HSC70

Flag-G6PD

β-Actin

HSPA8/HSC70

Flag-G6PD

HSPA8/HSC70

Flag-G6PD

β-Actin

**Figure S2A**

p-p70S6K (Thr389)

p70S6K

p-AKT(Ser473)

AKT

p-4EBP1

4EBP1

β-Actin

**Figure S2B**

p-p70S6K (Thr389)

p70S6K

p-AKT(Ser473)

AKT

p-4EBP1

4EBP1

β-Actin

**Figure S2C**

p-p70S6K (Thr389)

p70S6K

p-AKT(Ser473)

AKT

p-4EBP1

4EBP1

β-Actin

**Figure S3E**

p-mTOR (Ser2448)

mTOR

p-p70S6K

p70S6K

p-AKT(Ser473)

AKT

β-Actin

**Figure S3F**

p-mTOR (Ser2448)

mTOR

p-p70S6K

p70S6K

p-AKT(Ser473)

AKT

β-Actin

**Figure S4C**

PARP

Cleaved-PARP

β-Actin

PARP

Cleaved-PARP

β-Actin

Cleaved PARP

PARP

β-Actin

Cleaved PARP

PARP

β-Actin

**Figure S4H**

γ-H2AX

β-Actin

γ-H2AX

β-Actin

γ-H2AX

β-Actin

γ-H2AX

β-Actin

**Figure S4I**

p-ATM

p-CHK2

GAPDH

p-ATM

p-CHK2

GAPDH

**Figure S6F**

G6PD

GAPDH

G6PD

GAPDH

G6PD

GAPDH
